# Reference genome of the nutrition-rich orphan crop chia (*Salvia hispanica*) and its implications for future breeding

**DOI:** 10.3389/fpls.2023.1272966

**Published:** 2023-12-14

**Authors:** Parul Gupta, Matthew Geniza, Justin Elser, Noor Al-Bader, Rachel Baschieri, Jeremy Levi Phillips, Ebaad Haq, Justin Preece, Sushma Naithani, Pankaj Jaiswal

**Affiliations:** ^1^ Department of Botany and Plant Pathology, Oregon State University, Corvallis, OR, United States; ^2^ Molecular and Cellular Biology Graduate Program, Oregon State University, Corvallis, OR, United States

**Keywords:** chia, polyunsaturated fatty acid (PUFA), *Salvia hispanica*, biopeptide, reference genome, terpene synthase, lectins, seed mucilage

## Abstract

Chia (*Salvia hispanica L.*) is one of the most popular nutrition-rich foods and pseudocereal crops of the family Lamiaceae. Chia seeds are a rich source of proteins, polyunsaturated fatty acids (PUFAs), dietary fibers, and antioxidants. In this study, we present the assembly of the chia reference genome, which spans 303.6 Mb and encodes 48,090 annotated protein-coding genes. Our analysis revealed that ~42% of the chia genome harbors repetitive content, and identified ~3 million single nucleotide polymorphisms (SNPs) and 15,380 simple sequence repeat (SSR) marker sites. By investigating the chia transcriptome, we discovered that ~44% of the genes undergo alternative splicing with a higher frequency of intron retention events. Additionally, we identified chia genes associated with important nutrient content and quality traits, such as the biosynthesis of PUFAs and seed mucilage fiber (dietary fiber) polysaccharides. Notably, this is the first report of in-silico annotation of a plant genome for protein-derived small bioactive peptides (biopeptides) associated with improving human health. To facilitate further research and translational applications of this valuable orphan crop, we have developed the Salvia genomics database (SalviaGDB), accessible at https://salviagdb.org.

## 1 Introduction

Global-level crop improvement programs have focused mainly on cereal crops (rice, wheat, maize, millets), small grains, legumes, oilseed, and tuber crops. The genetic gains made through systematic breeding and translational research during the Green Revolution period and afterward had tremendous success in increasing grain production, quality, and yield that mitigated the problem of global hunger to a great extent in terms of calorie intake, and saved millions of lives (The Nobel Peace Prize, 1970; Swaminathan, 2009)—however, the issue of hidden hunger or malnutrition remains (Lowe, 2021). Thus, long-term food and nutrition security requires diversifying the human diet by incorporating produce from nutrient-rich minor crops. Minor crops are grown in limited quantities in geographically marginalized areas as part of low-input subsistence farming practices and were previously given little attention in breeding and genetic improvement programs, and thus, often referred to as "orphan crops" (Talabi et al., 2022). In recent years, the focus on the nutrient value of orphan crops, e.g., millets, chia, cassava, yam, small grains, and pulses, has increased global demands and expanded their consumer base. Their ability to grow in low-input marginal lands has become an important asset in mitigating the threats posed by global climate change (Zurita-Silva et al., 2014; Joshi et al., 2018; Woldeyohannes et al., 2020; Talabi et al., 2022). Thus, breeding high-yielding cultivars of orphan crops requires knowledge of candidate genes and genetic markers associated with important agronomic and nutrition traits. Investments in generating genomic resources for previously underutilized crops would help increase their production and the long-term sustainability of the agriculture and farming communities (Tadele and Assefa, 2012; Tadele and Bartels, 2019).

Chia (*Salvia hispanica* L.) is a minor crop primarily cultivated in Southern Mexico and Central America for its nutrient-rich seeds containing proteins, polyunsaturated fatty acids (PUFAs), dietary fibers, antioxidants, and minerals (Olivos-Lugo et al., 2010; Mohd Ali et al., 2012; de Falco et al., 2018; Kulczyński et al., 2019). Compared to dietary fiber sources like soybean, wheat, and maize, chia seeds contain approximately 54g/100g dietary fiber, of which ~93% is insoluble fiber (Alfredo et al., 2009). Similarly, 60% of all fatty acid comprises PUFA, and proteins comprise 18-24% of the seed mass (Kulczyński et al., 2019). Moreover, the health-benefiting effects of chia seeds (due to PUFAs) on improving muscle lipid content, cardiovascular health, total cholesterol ratio, triglyceride content, and anticarcinogenic properties have been demonstrated in humans and animals (Espada et al., 2007; Martínez-Cruz and Paredes-López, 2014; Marcinek and Krejpcio, 2017; Kobus-Cisowska et al., 2019; Kulczyński et al., 2019). Also, chia seeds' high dietary fiber content helps alleviate the hypoglycemic effect and stabilizes blood glucose levels in type-2 diabetic patients (Vuksan et al., 2007; Vuksan et al., 2010; Ho et al., 2013). Water-soaked chia seeds form a mucilaginous polysaccharide gel that acts as a texture modifier, emulsifier, gelling, and encapsulating agent in food, cosmetic, and pharmaceutical products (Chaves et al., 2018; Antigo et al., 2020; Chiang et al., 2021; Silva et al., 2022). Apart from the seeds, essential oils with reported antimicrobial activity (Elshafie et al., 2018) extracted from chia leaves are a rich source of secondary metabolites such as hydroxycinnamic acid derivatives, flavonoids, and sesquiterpenoids.

Chia is an annual herbaceous plant of the Lamiaceae (mint) family, which also includes popular culinary herbs. The genetic diversity, ploidy, and the number of chromosomes in the genus *Salvia* vary greatly from 2*n*=2*x*=12 in *S. hispanica* (chia) to 2*n*=8*x*=88 in octoploid *S. guaranitica (* Alberto et al., 2003; Palma-Rojas et al., 2017). Sequenced genomes of species from the genus *Salvia* include *S. miltiorrhiza* (Danshen, Chinese sage), *S. bowleyana* (Nan Danshen), *S. officinalis* (sage), *S. rosmarinus* (rosemary, *Rosmarinus officinalis*) and *S. splendens* (scarlet sage) (Dong et al., 2018; Song et al., 2020; Jia et al., 2021; Zheng et al., 2021; Li et al., 2022; Han et al., 2023). These members of the Lamiaceae family produce many unique secondary metabolites associated with human health benefits. For example, the Chinese sage genome (size 595 Mb) report suggests an expansion of secondary metabolism pathway genes, and the rosemary genome (size 1.11 Gb) contains carsonic acid biosynthesis pathway genes (Song et al., 2020; Han et al., 2023). The diterpene carsonic acid that accumulates in the leaf chloroplasts has antioxidant properties, and the rosemary plant is one of its richest sources (Richheimer et al., 1996; Loussouarn et al., 2017). The *S. bowleyana* genome (462 Mb) contains genes of the salvianolic acid B (Sal-B) biosynthesis pathway, and the *S. officinalis* genome (472 Mb) has extensive duplication of diterpenoid synthase gene family members (Zheng et al., 2021; Li et al., 2022). A handful of new genomic and transcriptomic studies for chia, including the recent reports of genome sequencing (NCBI; Wang et al., 2022; Alejo-Jacuinde et al., 2023; Li et al., 2023), are also discussed later. The current research on the reported chia is focused on genome sequencing, transcriptome analysis of important metabolic pathway genes (fatty acid metabolism, rosmarinic acid biosynthesis, and seed mucilage biosynthesis), and the identification of valuable genetic markers that can aid in crop improvement efforts (Kumari et al., 2015; Peláez et al., 2019; Gupta et al., 2021; Wang et al., 2022; Yue et al., 2022; Alejo-Jacuinde et al., 2023; Li et al., 2023).

**Figure 1:**
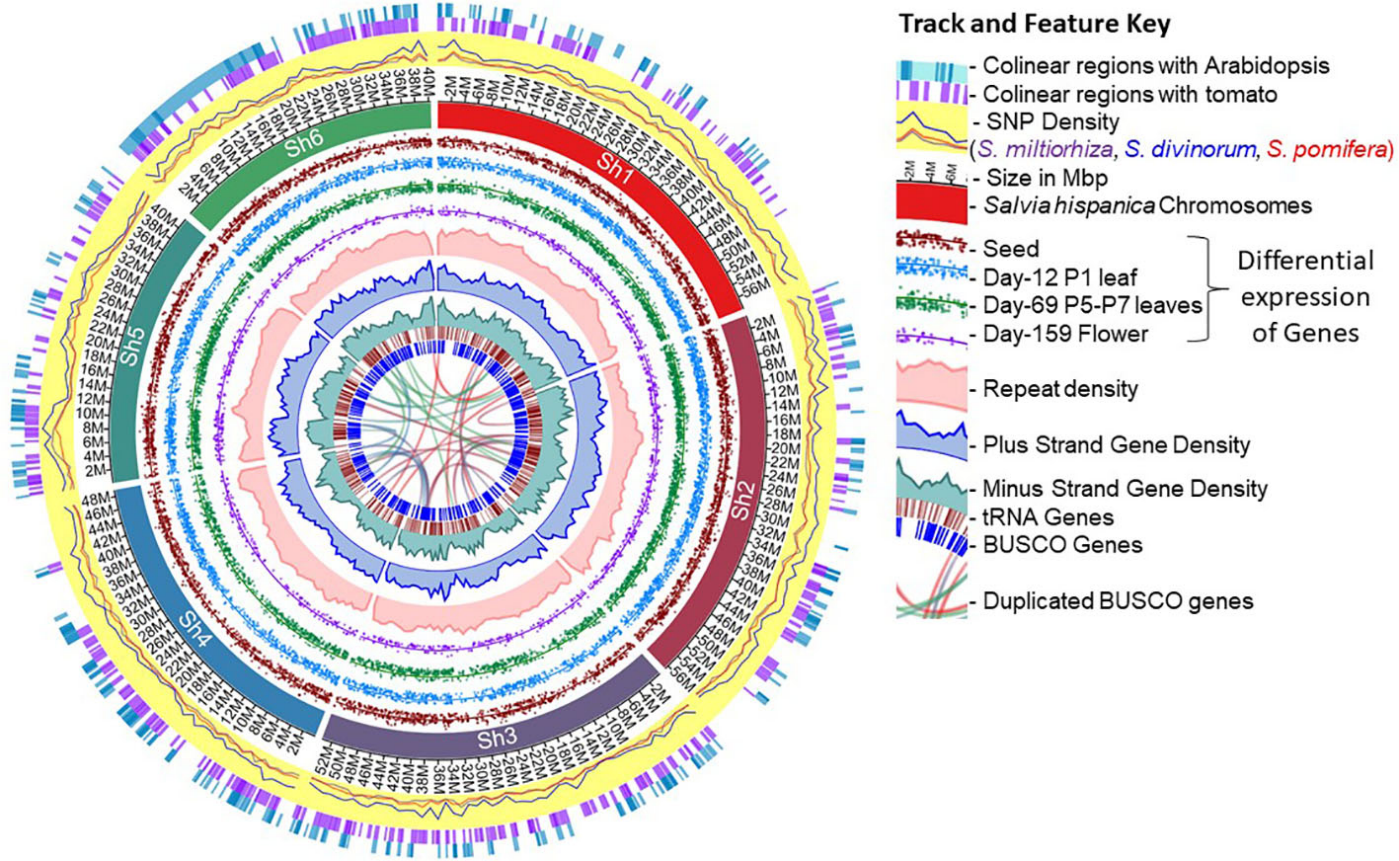
Overview of the chia (*Salvia hispanica*) genome assembly and the annotated features.

Here, we report a pseudochromosome-level chia reference genome assembly of 303.6 Mb with 48,090 annotated protein-coding genes. The gene models were supported by multiple sets of evidence, including mapped *de-novo* assembled transcript from the 13 different tissues and developmental stages of the chia plant (Gupta et al., 2021), homology with genes in other chia assemblies (NCBI; Wang et al., 2022; Li et al., 2023), the Viridiplantae-nr proteome set, and based on the protein domain presence. We also analyzed the gene models for genome-wide alternative splicing events. Furthermore, we conducted pathway enrichment analysis to characterize important genes involved in the biosynthesis of PUFA, monomer units of seed mucilage fiber, terpenes, and lectins. Finally, SNP marker analysis in the *Salvia* genus; and a comparison of genome synteny with Arabidopsis and tomato were conducted to explore the genome-level organization and changes (**Figure 1**).

## 2 Results

### 2.1 Genome sequencing, assembly, and scaffolding

We assembled the haploid genome of chia *with* an estimated genome size of ~356 Mb. Two short-read paired-end libraries with mean insert sizes ~363 bp and ~495 bp generated 416,408,443 and 551,676,481 raw read pairs, respectively. About 99% of the read pairs from each library passed the trimming procedure. K-mer based analyses of the reads estimated the nuclear genome size of ~356 Mb with k-mer size 109-mers and homozygous peak depth 175 (**Supplementary Figure 1**). *De novo* assembly of chia genome resulted in a final scaffold N50 of 32.9 Kbp, assembling 100% of the estimated non-repetitive genome size and 85.28% of the estimated genome size.

Further genome assembly and scaffolding improvements were made using the HiRise pipeline (Putnam et al., 2016). After collecting Chicago and Dovetail Hi-C data and scaffolding of the *de novo* assembly, we first used Chicago data plus HiRise, then used the resulting assembly as input into the HiRise pipeline along with the Dovetail Hi-C data. We improved the final scaffold N50 from 33 kb to 53.529 Mb (~1622-fold increase) and N90 from 7 kb to 40.353 Mb (~5765-fold increase). The final HiRise scaffolding resulted in 303.60 Mb of the assembled reference genome (~85% of the estimated genome size) with 2185 scaffolds with 2692.29x estimated physical coverage (**Table 1**). The coverage values were calculated as the number of read pairs with inserts between 10 to 10,000 kb spanning each position in the input assembly. The six largest scaffolds representing the haploid chromosomes (*x*=6) or pseudomolecules comprised 299.03 Mb of the sequenced genome. The largest scaffold length is 57,938,346 bp.

**Table 1:** Genome assembly and annotation summary.

Genome assembly and scaffolding	
Description	Counts or Length
Estimated genome size	356.1 Mb
Sequenced genome size	303.6 Mb (303,603,087 bp)
Number of scaffolds	2185
Number of scaffolds >1 kb	2185
Number of scaffolds >40 Mb	6
Scaffold L50/N50	53.529 Mb
Scaffold L90/N90	40.353 Mb
Cumulative size of 6 largest scaffolds	299.03 Mb
Number of Gaps	19891
Percent of the genome in gaps	0.65%

Genome assembly and scaffolding	
Description	Counts or Length
Size of six assembled chromosomes	299,036,396 bp
Percent repeat masked	42.09%
Number of protein-coding (CDS) genes	48,090
Number of tRNA genes	799
Number of rRNA genes	37
Number of transcripts (mRNAs)	54,503
Mean gene length	2041 bp
Longest gene length	44,939 bp
Shortest gene length	201 bp
Mean exon length	204 bp
Number of genes with a single exon	9,776
Number of genes with multiple exons	38,314
Chloroplast genome size (complete)	151,778 bp
Number of chloroplast genes	157
Mitochondrial genome size (partial)	302,349 bp
Number of mitochondrial genes	103

### 2.2 Genome quality and integrity

Almost all (99%) of the *de novo* assembled transcripts derived from our recently published transcriptome atlas dataset from 13 tissues (Gupta et al., 2021) (**Table 2**) mapped on the six largest scaffolds, suggesting that these six largest scaffolds cover almost all the transcribed regions and represent the six haploid chromosomes. The chromosomes were named Sh1-6 in the descending order of their bp length (**Supplementary Table 1**). Additional benchmarking of the completeness of the gene space in the assembled genome using the BUSCO analysis (v 5.3.2) revealed 97.6% of the Viridiplantae representation and about 96% for the eudicot set, suggesting a high percentage of the gene space captured in the assembled chia genome (**Supplementary Table 2**).

**Table 2:** Description of the chia plant samples used for generating the transcriptome atlas, published earlier (Gupta et al., 2021).

Growth stage	Sample collection days after sowing (DAS)	Sample description	Sample name
Vegetative	Day 0	Dry Seed	Seed
	Day 3	Green cotyledon	D3-Cotyledon
	Day 3	Above ground shoot parts (whole shoot)	D3-Shoot
	Day 12	Above ground shoot parts (whole shoot)	D12-Shoot
	Day 12	Very first/youngest leaf at the shoot apex	D12-P1
	Day 69	First and second leaves at the shoot apex	D69-P1-P2
	Day 69	Third and fourth leaves at the shoot apex	D69-P3-P4
	Day 69	Fifth, sixth, and seventh leaves at the shoot apex	D69-P5-P6-P7
	Day 69	Internode between the 5^th^ and 6^th^ leaves	D69-Internode
Reproductive	Day 158	The top half of the raceme inflorescence (pre-anthesis)	D158-RacemeTopHalf
	Day 158	The bottom half of the raceme inflorescence (with pre-anthesis flowers)	D158-RacemeBottomHalf
	Day 159	Flowers from Day-1 of flowering (anthesis stage)	D159-Flowers
	Day 164	Flowers from Day-5 of flowering (anthesis stage)	D164-Flowers

### 2.3 Organelle genomes

Complete assembly of the plastid genome resulted in a single contig of 151,778 bp length made from ~35M overlapping reads and contains 157 genes (**Supplementary Figure 2A**). In contrast, the mitochondrial genome assembly resulted in a partial genome of 302,349 bp based on ~18M reads and contains 103 genes (**Supplementary Figure 2B**). We also observed 19 regions on the six chia chromosomes (Sh1-6) that show >98% identity to the plastid genome features with an average length of ~2400 bp, the longest being 4,328 bp long (**Supplementary Figure 2C**). Similarly, 20 such events of mitochondrial origin were identified with an average length of ~2,000 bp, the longest being a 5,704 bp feature (**Supplementary Figure 2D**). No mitochondrial genome features were observed on chromosome Sh4.

### 2.4 Gene model prediction and functional annotation

The genome assembly was repeat masked by identifying its repeat content that accounts for 42.09% of the chia genome. The repeat elements were classified as LTR retroelements (18.24 Mb), DNA elements (5.8 Mb), and LINEs (0.9 Mb). However, the most abundant repeat sequences (99.63 Mb) were unclassified, those unavailable in public databases (**Supplementary Table 3**). We only used the six pseudomolecules (Sh1-6) for gene model prediction and downstream analysis. A masked genome was used to predict gene models using AUGUSTUS (Hoff and Stanke, 2018). A total of 48,743 protein-coding genes were predicted, which were filtered using gFACs (Caballero and Wegrzyn, 2019) to obtain non-redundant and complete gene models (including start and stop codons). Additionally, we incorporated evidence from the presence of conserved protein domains, mapping *de-novo* assembled transcript (Gupta et al., 2021), and homology with genes in other chia genome assemblies (NCBI; Wang et al., 2022; Li et al., 2023) and the Viridiplantae-nr proteome set that resulted in identifying 48,090 protein-coding genes (**Table 1**). The mean size of the predicted protein-coding genes was 2041 bp (**Table 1**). There were 9,776 mono-exonic and 38,314 multi-exonic genes, and the average exon length was 204 bp compared to 340 bp for intron. The largest intron size was 16,979 bp.

Functional annotation analysis showed that of the 48,090 predicted gene models (one per loci), 33,710 were annotated using InterProScan (Jones et al., 2014). A total of 18,620 proteins were assigned to Gene Ontology (GO) terms, and 3,125 were annotated using Pfam (**Supplementary Table 4**). We used TargetP (Almagro Armenteros et al., 2019) and TMHMM (Krogh et al., 2001) to predict the subcellular locations of the proteins encoded by the chia genome. A total of 7,455 proteins have signal peptide sequences for potential localization to the endoplasmic reticulum, chloroplast, thylakoid lumen, and mitochondria (**Supplementary Figure 3A**). The 9,961 proteins have one or more transmembrane domains (**Supplementary Figure 3B**) predicted by the TMHMM (Krogh et al., 2001). 31,847 gene models were annotated using BLASTP (Camacho et al., 2009) against the NCBI-Viridiplantae database (**Supplementary Table 5**).

The chia genome has 799 tRNA genes, which is 30 and 70 percent more genes compared to the tomato and Arabidopsis genomes, respectively. Also, tRNA genes corresponding to the codons GUC (valine) and AGU (serine) are unique to chia (**Supplementary Table 6**). GUC represents about 20% of all valine codons present in the coding sequences of all chia proteins. At the same time, AGU represents 15% of all serine codons in the coding sequences of all chia proteins. The ribosomal RNA (rRNA) annotation identified 37 rRNA genes in the genome (**Supplementary Table 7**). Of these, only ten are present on the pseudochromosomes. At least one 45S subunit gene cluster on chromosome Sh6 carries the components 18S and 25S within a 5kbp region.

### 2.5 Comparison to other sequenced chia genomes

We compared the recently published reports of *S. hispanica* genome sequences to our chia genome assembly and gene mappings. As shown in **Supplementary Figure 4**, the synteny analysis between previously reported chia genomes by Wang et al. (2022), Li et al. (2023), and the assembly from the University of Melbourne (UniMelb) (NCBI) suggest significant (and somewhat unreconcilable) differences in gene mappings on the chromosomes. However, 95-98% of all genes identified in these assemblies map to our genome assembly, whereas 96-99%of our genes map on their genomes. Our genes map to 88-94% of their genes, whereas only 77-87% of their genes map to our genes. Of the 48,090 genes in our assembly, 22,925 genes were common to all assemblies, and only 5,297 were unique to our annotations (**Supplementary Figure 4**). The previously published *de-novo* assembled transcripts (Gupta et al., 2021) support 86% of our genes and 82-92% of their genes. Almost all *de-novo* transcripts map to all the genome assemblies. Ours and Wang et al. (2022) assemblies have similar 42% and 47% repeat content, respectively, compared to the 54% reported for the Li et al. assembly (Li et al., 2023). The genome assembled in the six pseudo-chromosomes in our (Shotgun-based, 299 Mb) and the UniMelb (long-read; 297 Mb) assemblies are very similar, compared to the larger Li et al. (2023) 362 Mb and Wang et al. (2022) 348 Mb chromosome-level assemblies (**Supplementary Figure 5**).

### 2.6 Chia genome shares greater synteny with tomato

**Figure 2:**
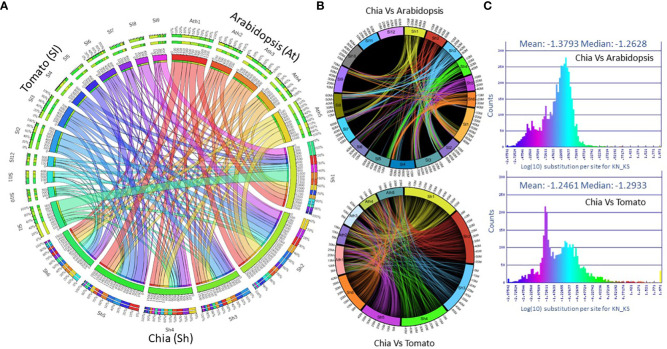
Synteny analysis of the chia genome. **(A)** Chromosome wise colinear genes shared between chia, Arabidopsis and tomato; **(B)** Chromosome wise collinear gene location; **(C)** The ratio of non-synonymous (Kn) to synonymous (KS) substitutions per site between syntenic orthologues.

The synteny analysis with the *Arabidopsis thaliana* and tomato (*Solanum lycopersicum*) genomes found 2,282 (2.35%) and 7,334 (8.9%) colinear genes, respectively (**Figure 2A**). We identified 138 colinear genome regions between chia and Arabidopsis, compared to the 336 regions between chia and tomato. Based on the colinear genes, chia chromosome Sh1 shows synteny with tomato chromosomes SL1, 4, and 9. Sh2 is syntenic with SL2, 3, 6, and 8, Sh3 is syntenic with SL2 and 4, Sh4 with SL7 and 12, Sh5 with SL2, 3, 5, 6, and Sh6 with SL3, 6, 7 and 11 (**Figure 2B**). When compared to Arabidopsis, chia shows widespread syntenic regions across Arabidopsis chromosomes (**Figure 2B**). The log10 ratio test of the non-synonymous (Kn) to synonymous (Ks) substitutions per site between syntenic orthologs between chia and tomato is -1.246. In contrast, Arabidopsis is -1.379, suggesting higher similarity with tomato (**Figure 2C**).

### 2.7 Gene expression atlas and alternative splicing survey

After removing genes with low read counts, a total of 34,885 genes were used for differential gene expression analysis, of which 20,340 genes show a significant transcript abundance difference (FDR <0.05, Log2 fold change >1.0) when compared to at least one tissue sample (**Supplementary Table 8**). **Supplementary Table 8** summarizes differentially expressed genes (DEGs) across all tissues mentioned in **Table 2**. Mature leaves (D69-P5-P6-P7) contain a maximum number (4,986) of DEGs, followed by seed (4,975), young leaves (D12-P1) (4,846), internode (4,712), and cotyledons (4,695). The seed sample contains a maximum number (1,719) of upregulated (log2 FC ≥ 2) genes, and mature leaves (D69-P5-P6-P7) have a maximum number (3,585) of downregulated genes (log2 FC ≤ -2) (**Supplementary Table 8**). Interestingly, for each tissue type, the number of downregulated genes is higher than the upregulated genes.

The expression atlas was analyzed for profiling the alternative splicing of the transcripts. We created highly accurate splice site classifiers with canonical (GT-AG) and semi-canonical (GC-AG) splice sites to filter the splice junctions in RNA-Seq read alignments. We observed ~99.1% of GT-AG and ~0.87% GC-AG splice junctions. After removing the false positive splice sites, we found 42,909 splicing events categorized as intron retention (IR), exon skipping (ES), alternative 5' splicing (Alt5'), and alternative 3' splicing (Alt3') events. These events were observed in 21,291 genes (~44%) (**Supplementary Table 9**). Chromosome Sh2 has the most spliced genes; the least was on Sh6. IR appeared as the most common event, followed by ES, Alt3' and Alt5' events. A total of 10,852 genes are differentially expressed and spliced (DAS).

### 2.8 Gene family and phylogenetic analysis

**Figure 3:**
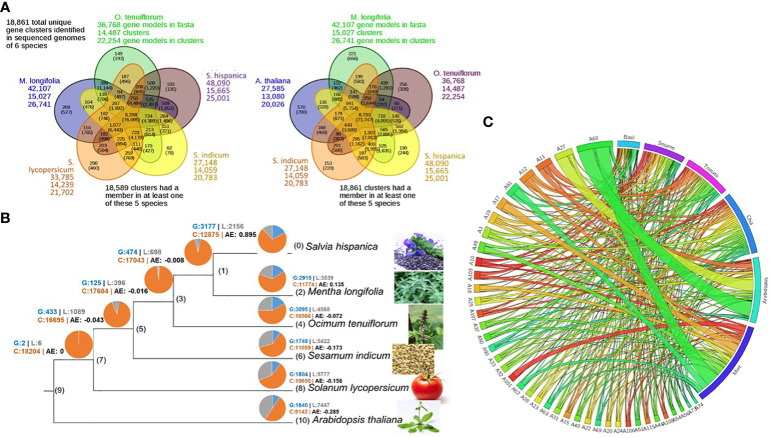
Chia gene family and phylogenetic analysis. **(A)** Inparanoid-based gene family analysis; **(B)** Gene family member gain/loss analysis. Gains (blue), conserved (orange), and loss (Grey); **(C)** Species wise gene distribution in the most significant families.

The five closely related species, basil (*O. tenuiflorum*), mint (*M. longifolia*), chia (*S. hispanica*), sesame (*S. indicum*), and tomato (*S. lycopersicum*) and the outgroup Arabidopsis (*A. thaliana*), share 18,861 gene family clusters with at least one or more of the six species represented in each family. Of these, 9,298 genes represent the common core set, with 76,095 genes from all six species. About 25,000 (58%) chia genes are members of 15,665 gene families (**Figure 3A**).

**Figure 4:**
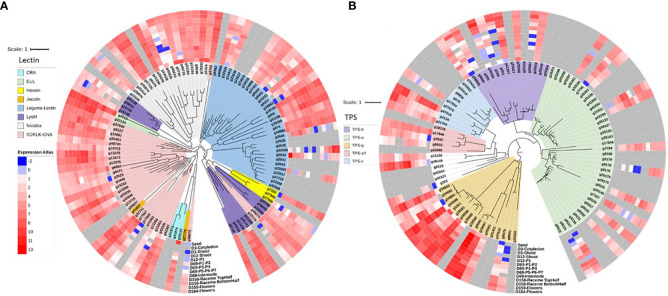
Chia gene family and expression analysis. **(A)** Lectin gene family tree with gene expression profiles; **(B)** terpene synthase (TPS) gene family tree with gene expression profile data.

The gene gain and loss analysis among the six species suggests that the chia genome has the highest number of conserved genes (12,875), and as a representative of the genus *Salvia*, it gained 3177 genes and lost 2156 genes. The average expansion (AE) rate of gene gain vs. loss was 0.895 compared to 0.135 for mint (**Figure 3B**). Distribution of the most significant gene families across six species was also analyzed (**Figure 3C**). The lectin and terpene synthase gene families were analyzed in detail among the many significant gene families. We identified 98 members of the lectin family homologs in chia based on sequence similarity to Arabidopsis lectin family members described previously by Naithani et al. (2021b). About 24% of lectin gene family members occur as tandem duplicates in the chia genome and show variability in their domain structure and tissue-specific gene expression profile. Compared to Arabidopsis, chia shows differences in the number of closely related orthologs. For example, we found 16 Lysin motif-containing lectins (LysM)-coding genes in chia corresponding to 14 in Arabidopsis. LysMs are known to bind GlcNAc-containing molecules produced by bacteria (i.e., lipo-chitooligosaccharides, chitooligosaccharides, and peptidoglycan) and play a role in activating plant immunity responses, including the synthesis of secondary metabolites with antimicrobial activity (Buendia et al., 2018; Naithani et al., 2021b). Also, chia has two Euonymus lectin (EUL) coding genes as opposed to one in the Arabidopsis. EUL genes are known to be differentially regulated in response to various stress conditions and are likely to play a role in cell signaling and defense response (De Schutter et al., 2017). The legume-lectin-like and those containing SDRLK-GNA domains were the largest sub-families. Homologs of the two Arabidopsis Ricin-B lectins were absent in the chia genome. Other members represent the class V chitinase-related agglutinin (CRA), Hevein, Nictaba, legume-lectin, and Jacalin subfamilies (**Figure 4A**). A number of these family members have high transcript abundance in the mature chia seeds, like the legume-lectins (g2634, g32505, g37451), Jacalin (g26137), EULs (g2516), and Nictaba (g665, g19165, g1424, g19233, g5726, g40776).

Similarly, homology and protein domain-based searches identified 90 terpene synthase (TPS) gene family members. The members were distributed in the previously identified subfamilies TPS-a, TPS-b, TPS-c, TPS-e/f, and TPS-g (Myburg et al., 2014). All sub-families have at least one gene member highly expressed in the seed, except for the TPS-a members (**Figure 4B**). Several TPS genes show higher expression in reproductive plant parts and leaves at various stages of development.

### 2.9 Metabolic pathway analysis

Pathway annotations were assigned to 10,039 chia genes by homology-based comparisons to Plant Reactome (Naithani et al., 2020) and KEGG (Kanehisa et al., 2016) pathway databases. These annotations allowed us to explore the biosynthesis pathways of fatty acid, PUFA, and seed mucilage monomer units in detail. Long-chain fatty acids serve as the building blocks for PUFAs. We identified genes for each step of the fatty acid biosynthesis process and analyzed the expression patterns of mapped chia genes. We identified 49 genes for this pathway, of which 42 were differentially expressed and 35 underwent splicing. Only 29 genes show DAS (differentially expressed and spliced) profile. For the fatty acid biosynthesis pathway (**Supplementary Figure 6**), we found eight genes encoding for different subunits of acetyl-CoA carboxylase (EC 6.4.1.2), responsible for catalyzing acetyl-CoA to malonyl-CoA. Acetyl-CoA carboxylase is a multi-subunit enzyme composed of four different polypeptides, biotin carboxyl carrier protein (accB), biotin carboxylase (accC), α-carboxyltransferase (accA), and β-carboxyltransferase (accD) (Cronan and Waldrop, 2002). Two genes (g10031, g23582) code for accB, three genes (g15971, g18937, g39581) code for accC, and two genes (g8594, g8614) code for accA, and gene g1426 code for accD. In the following steps, the malonyl group from malonyl-CoA is transferred to acyl carrier proteins (ACPs), followed by elongation of the acyl chain up to 16 or 18 carbons. At the last step of the fatty acid biosynthesis pathway, two types of Acyl-ACP thioesterase enzymes (FATA and FATB) (EC 3.1.2.14) serve as a determining factor for generating a variety of fatty acids in an organism. We identified one FATA gene (g22614) and three FATB genes (g32094, g43474 and g44663). Except for 11 genes that are expressed in seeds, most of the fatty acid biosynthesis genes are highly expressed in early-stage vegetative tissues and reproductive stages but not in seeds (**Supplementary Figure 6**).

### 2.10 PUFA biosynthesis pathway

**Figure 5:**
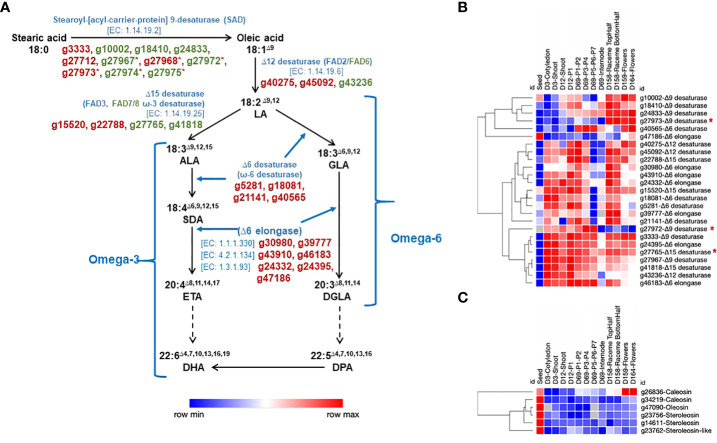
Chia genes involved in PUFA biosynthesis pathway and their expression across different developmental stages. **(A)** Biosynthesis of omega-3 and omega-6 fatty acids. LA, Linoleic acid; ALA, α-linolenic acid; GLA, γ‐linolenic acid; SDA, Stearidonic acid; ETA, Eicosatetraenoic acid; DGLA, dihomo-γ-linolenic acid; DHA, Docosahexaenoic acid; DPA, Docosapentaenoic acid. **(B)** expression of genes involved in omega-3 and omega-6 fatty acids biosynthesis; **(C)** expression of oleosin, caleosin, and steroleosin genes. The reactions with dashed arrows represent multiple steps. The gene IDs marked with an asterisk (*) represent tandemly duplicated genes. Gene IDs and names in green text are potentially targeted to chloroplast.

We identified 29 genes involved in the PUFA biosynthesis, including genes responsible for catalyzing the desaturation of stearic acid (SA) (18:0) to omega-3 and omega-6 fatty acids, some of which also occur in the chloroplast (**Figure 5A**). Of a total of 29 genes, 23 were differentially expressed, 17 spliced, and 14 showed DAS profile. Desaturation of stearic acid begins at C-9 position (Δ9) and further extends to α-linolenic acid (ALA) (18:3^Δ9,12,15^) in a three-step process. The first step involves the conversion of SA to oleic acid (18:1^Δ9^) catalyzed by Stearoyl-[acyl-carrier-protein] 9-desaturase (SAD) (EC 1.14.19.2), encoded by at least 11 genes (g3333, g10002, g18410, g24833, g27712, g27967, g27968, g27972, g27973, g27974, g27975). Six of these tandemly duplicated genes are on chromosome Sh3. Seven SAD genes showed varied expression profiles across various developmental stages; however, four SAD genes did not express in any tissue type (**Figure 5B**). Of 11 SADs, 7 contain transit sequence for chloroplast localization, whereas 4 SADs showed endoplasmic reticulum (ER) localization signal. The Δ12 desaturase (EC 1.14.19.6) catalyzes the conversion of oleic acid (18:1^Δ9^) to linoleic acid (LA; 18:3^Δ9,12^). We identified three Δ12 desaturase genes, of which two showed ER, and one showed chloroplast localization signal. The Δ15 desaturase enzyme (EC 1.14.19.25) catalyzes the conversion of LA to ALA. We identified four genes encoding this enzyme, two contain ER (g15520, g22788), and two have chloroplast (g27765, g41818) transit signals. In addition, we identified four potential candidate Δ6 desaturase genes (g5281, g18081, g21141, g40565) responsible for the conversion of stearidonic acid (SDA- 18:4^Δ6,9,12,15^) from ALA and γ‐linolenic acid (GLA-18:3^Δ6,9,12^) from LA. Homology-based prediction of Δ6 desaturases showed ~75% identity with evening primrose desaturase protein sequences (Fu et al., 2017). Primrose and our Δ6 desaturase proteins share highly homologous regions with higher plant Δ8 desaturases (Fu et al., 2017). Further elongation of SDA and GLA is catalyzed by an Δ6 elongase enzyme complex, which consists of different enzyme activities, a β-ketoreductase, a dehydrase, and an enoyl reductase (Beaudoin et al., 2000). Homology-based prediction helped identify seven putative Δ6 elongase genes (**Figure 5A**). In contrast to our earlier transcriptome atlas study, the genome annotation enabled the identification of Δ6 desaturase and elongase candidate genes (Gupta et al., 2021).

The lipid-rich plant seeds often contain oil bodies (OBs) called oleosomes, considered cellular organelles (Shao et al., 2019). The neutral lipids stored within the OBs serve as an essential source of energy and carbon required for supporting the seed germination phase and early seedling development. The OBs are also involved in cellular processes such as response to stress, lipid metabolism, phytohormone signaling, and development. The function of seed OBs in plants, including chia, depends on membrane-specific oleosin, caleosin, and steroleosin structural proteins, localized to the OB phospholipid monolayer (Lin et al., 2002; Lopez et al., 2023). Three genes encoding for oleosin (g23053, g45274, g47090), three for caleosin (g26836, g27493, g34219), two for steroleosin (g14611, g23756), and three for steroleosin-like (g9498, g23762, g30197) proteins were identified in the chia genome. Of these, one oleosin, two caleosin, and both steroleosin genes show higher expression in seed (**Figure 5C**).

### 2.11 Seed mucilage biosynthesis pathway

**Figure 6:**
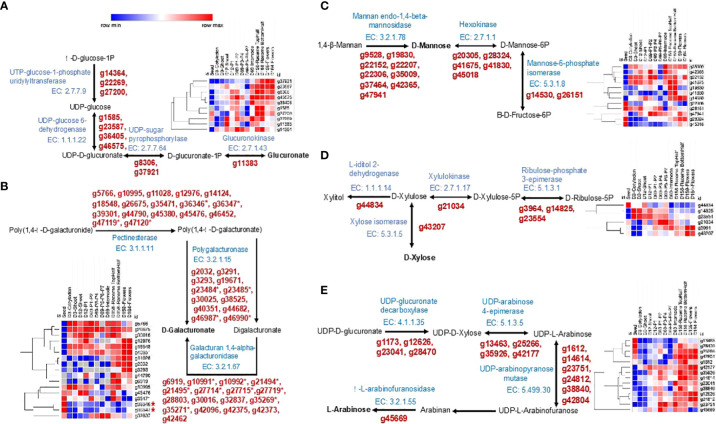
Biosynthesis pathways and expression analysis of Chia genes involved in biosynthesis of seed mucilage polysaccharide monomer units. **(A)** Glucuronate biosynthesis; **(B)** D-Galacturonate biosynthesis; **(C)** D-Mannose biosynthesis; **(D)** D-Xylose biosynthesis; **(E)** L-Arabinose biosynthesis. The gene IDs marked with an asterisk (*) represent tandemly duplicated genes.

Chia seeds are an excellent source of dietary fibers containing ~40% of fiber, 5–10% of which is soluble fiber and forms part of the mucilage (Reyes-Caudillo et al., 2008; Tavares et al., 2018; Khalid et al., 2023). Water-soaked chia seeds produce a mucilaginous polysaccharide around them. This fiber-rich mucilage has wide applications in pharmaceutical, food, and cosmetic industries (Antigo et al., 2020; Chiang et al., 2021; da Silveira Ramos et al., 2021). It consists of heteropolysaccharides composed of D-xylose, D-glucose, D-mannose, L-arabinose, galacturonic, and glucuronic acid residues (Lin et al., 1994; Timilsena et al., 2016; da Silveira Ramos et al., 2021). We identified 93 genes catalyzing basic monomer units of polysaccharides of chia seed mucilage. Of them, 45 were differentially expressed, 36 underwent splicing, and 20 showed a DAS profile. Most of the genes of glucuronate monomer biosynthesis pathway show lower expression in seed except two genes (g22269, g27200) encoding for UTP-glucose-1-phosphate uridylyltransferase (**Figure 6A**). Of the total ten genes involved in the glucuronate biosynthesis, five genes (g8306, g22269, g14364, g37921, g27200) are subjected to splicing. A total of 46 genes mapped to the D-Galacturonate monomer biosynthesis, of which 12 genes (g45380, g46987, g2032, g10991, g10992, g19671, g44790, g45476, g3293, g6919, g11028, g18548) undergo splicing. Five genes (g10955, g35471, g36346, g36347, g45476) encoding pectinesterase (EC 3.1.1.11) are highly expressed in seed (**Figure 6B**). Genes g36346, g36347 are tandemly duplicated on chromosome Sh6. For the D-Mannose monomer biosynthesis pathway, we identified 16 genes, of which six genes (g9528, g20305, g42365, g45018, g22152, g22306) show splicing. Five genes (g22306, g26151, g47941, g28324, g45018) are highly expressed in the seed (**Figure 6C**). Synthesis of D-Xylose from D-Xylulose is catalyzed by xylose isomerase (EC 5.3.1.5), encoded by g43207 showing higher expression in reproductive developmental stages and seed but very low expression in D3 developmental stages (**Figure 6D**). The L-Arabinose monomer biosynthesis pathway consists of 4 reactions catalyzed by enzymes, EC 4.1.1.35, EC 5.1.3.5, EC 5.499.30, 3.2.1.55, and one spontaneous reaction (Arabinan from UDP-L-Arabinofuranose). A total of 15 genes were identified for this pathway and of which 14 were expressed across different developmental stages (**Figure 6E**). Nine genes (g42177, g1173, g14614, g38840, g42804, g45669, g1612, g28470, g13463) undergo splicing. At least one gene in each enzymatic step is highly expressed in seed and reproductive organs.

### 2.12 Exploring small bioactive peptides in the chia proteome

The dietary proteins that are part of our food intake are digested and hydrolyzed into small peptides and amino acids by the proteases (proteolytic enzymes), for example, pepsin, trypsin, chymotrypsin, elastase, and carboxypeptidase, acting in the gastrointestinal tract. These biopeptides are then absorbed by the intestinal tract and transported via blood circulation for use in the human body. Many of the small peptides with 2-60 amino acids generated in the process (also known as biopeptides) are known to play biological roles other than simply serving as the protein component of the diet (Daliri et al., 2017). Many biopeptides derived from plant proteins (including those from chia) are known for their potential pharmaceutical applications in improving human health (Orona-Tamayo et al., 2015; Grancieri et al., 2019; Peláez et al., 2019; San Pablo-Osorio et al., 2019; Aguilar-Toalá and Liceaga, 2020; Patil et al., 2020; Morales et al., 2021; Rabail et al., 2021; Aguilar-Toalá et al., 2022). The studies suggested that individual biopeptides or biopeptide complexes (a mixture of several biopeptides) contain antidiabetic, anorectic, anti-hypertension, antimicrobial, hypocholesterolemic, anti-oxidative and anti-skin aging (anti-wrinkle) activities (Orona-Tamayo et al., 2015; Cotabarren et al., 2019; Patil et al., 2020).

We conducted an *in-silico* analysis of the annotated chia proteome against the curated library of about ~4000 known bioactive peptides (many from animal origin) and their reported roles in human health (**Supplementary File 1**). We found about 6 million instances of 697 biopeptide types with known Angiotensin Converting Enzyme inhibitor (ACE-inhibitor) in the chia proteome. ACE-inhibitors are a class of medication used to treat hypertension and cardiovascular conditions (Herman et al., 2022). Similarly, we found ~8.6 million instances for 322 known biopeptides with antidiabetic Dipeptidyl peptidase 4 (DPP-4) inhibitor roles. DPP-4 inhibitors are a group of antihyperglycemic medications used to manage type-2 diabetes. The proteins encoded by 2707 differentially upregulated genes (≥1.0) in the edible seed samples carry biopeptides associated with 18 different types of roles in alleviating many diseases, including type-2 diabetes (DPP-4), high blood pressure (ACE inhibitors, Renin inhibitor), antiaging, antiviral, immunomodulating, etc. (**Supplementary File 1**). These include proteins with seed storage and oxidoreductase function that carry the antiaging peptides. Three Nictaba and one legume lectin domain-containing proteins have peptides with known roles regulating the stomach mucosal membrane activity and phosphoinositol metabolism (**Supplementary File 1**).

### 2.13 SNP and SSR markers

The SNP analysis using reads from seven different *Salvia* species and two Salba chia variants (black and white) identified about 3 million (3,027,193) unique SNP sites, including indels, in the reference chia genome. About 9% of the sites (339,762) carry SNPs from multiple samples. *S. splendens* has the most SNPs with 1,836,204 sites, compared to 854,877 in *S. divinorum*, 358,708 in *S. pomifera*, 344,540 in *S. miltiorrhiza*, about 11,000-12,000 sites were common with salba's and a small proportion in the *S. officinalis*, *S. rosmarinus* and *S. yangii* (**Supplementary Table 10**). Most of the SNP substitution types were purine to purine (A↔G) and pyrimidine to pyrimidine (C↔T) transitions (Ti) compared to the purine to pyrimidine or vice versa (A↔T, G↔C, G↔T, A↔C) transversions (Tv). *S. officinalis*, *S. rosmarinus*, and *S. yangii* have a significantly lower number of transitions G→A and C→T sites. The transversion sites, G→C, and C→G were least represented in all except in *S. pomifera* where G→T and C→A were least represented. Black is more like the reference genome when compared between the Salba genotypes and carries fewer SNPs than white. The Ti/Tv ratio of 1.69 was highest for *S. pomifera*, followed by *S. divinorum* (1.62) and *S. miltiorrhiza* (1.59). *S. splendens*, with 72,026, and *S. divinorum*, with 6703, have the most indel sites. In the assembled chia reference genome, the search for 2-6 mer long simple sequence repeat (SSR) motifs identified 15,380 sites as potential genetic markers on the six chromosomes (**Supplementary Table 11**). Of these, we found 7,988 dimers, 5,699 trimers, 800 tetramers, 309 pentamers, and 584 hexamers. The average distance between SSRs is 2,866 bp.

### 2.14 *Salvia* genome database

Salvia Genomics Database (SalviaGDB, https://salviagdb.org) was developed as a web-accessible community resource to allow researchers to identify all the *Salvia* genus-related genomic datasets. We preserved the original annotations from the source providers (Dong et al., 2018; Song et al., 2020; Jia et al., 2021; Zheng et al., 2021; Li et al., 2022; Wang et al., 2022; Han et al., 2023; Li et al., 2023). Additional data integration as new data tracks on the reference genome includes transcriptome sequence alignments, SNPs, expression data, and Minimap-based genes and transcript liftoff. We will soon add the synteny, gene homology, InterProScan, and ontology-based functional annotation of genes.

## 3 Discussion

With the advent of improved high-throughput sequencing technology and platforms, it became possible to obtain the genome sequences for multiple accessions of the same species and/or expand the plant genomic studies to include minor crops and other species. The availability of closely related sequenced genomes aids in understanding the conserved regions among the members of the same taxonomic clade and the accessory genetic content and sequence variations associated with important agronomic and evolutionary traits. Here we report a chromosome-scale genome assembly of chia (dicotyledonous, diploid plant 2*n*=2*x*=12 with six pairs of chromosomes (Estilai et al., 1990; Ranjbar et al., 2015)) by integrating Illumina and Dovetail's Hi-C and Hi-Rise technologies. This assembled chia genome is ~303.6 Mb in size and consists of 2185 scaffolds with N50 of 53.529 Mb (see **Table 1**), representing about 85.25% of the estimated genome size (~356 Mb).

We have compared our chia genome assembly and its annotations to the previously published chia genome sequences (NCBI; Wang et al., 2022; Li et al., 2023). The sizes of the six assembled chromosomes reported in this study are similar to the long-sequence read-based Australian UniMelb assembly (NCBI). Chia genome assembly by Wang et al. (2022) is of larger size (348 Mb) than the chia genome reported by our group. However, the reported repeat masked region between the two assemblies is approximately similar (45% vs. 42%). Another chia genome produced using long-read sequencing accounted for 361.7 Mb in size with a repeat masked region of 54% by Li et al. (2023). We speculate that some long-read sequence-based assembly by Li et al. (2023) successfully captured the non-gene-enriched repetitive sequences reflecting the larger genome size. Our chia genome assembly contains ~94% of the gene space as per the BUSCO analysis (**Supplementary Table 2**) and covers 96-98% of the gene space with 48,090 protein-coding genes, which is higher than the chia genes in the .tudies mentioned above. Some increase in gene numbers in our report can be explained due to the differences in the genome sequencing and gene annotations methods used. The difference in the genome size vs. the sequencing methods is very apparent. Here we note the details of gene annotation methods implemented in different studies. Wang et al. (2022) based chia gene annotations used in-house generated transcript data from chia roots, stems, leaves, and flowers of four-month-old plants, and additional support from orthologous gene models from *Arabidopsis thaliana*, *Solanum lycopersicum*, *Sesamum indicum*, *S. miltiorrhiza*, and *S. splendens*. On the other hand, Li et al. (2023) used the chia RNA-seq data previously published by our group (Gupta et al. (2021)) and support from orthologous polypeptide sequences from *A. thaliana*, *Antirrhinum majus* (version IGDBV1), *Callicarpa americana*, *S. miltiorrhiza* (version 1.0), *S. splendens*, *Tectona grandis* and the UniProtKB/Swiss-Prot dataset (version release-2020_04). Li et al. (2023) did not use any transcriptome data generated from the same accession of chia they used for genome sequencing.

In our study, we like to highlight that the same chia accession was used for genome sequencing and exhaustive transcriptome sequencing (from 13 tissue types, including seeds, cotyledon, shoots, leaves, internodes, racemes, and flowers) (Gupta et al., 2021). Thus, the higher transcript support for gene models in this study may reflect the identification of more gene loci in our genome assembly compared to those identified by Li et al. (2023). The fewer protein-coding genes reported by Wang et al. (2022) may also account for using relatively less enriched transcripts set sampled at a single developmental stage (4-month-old plants) than our transcript datasets. It is crucial to emphasize that genome assembly and gene annotations improve as advancements in sequencing technology and bioinformatics pipelines occur. For instance, the genomes of major model organisms, such as humans, Arabidopsis, rice, and maize, are continually improving. Likewise, we expect continuous improvement in the reference chia genome assembly and gene annotations.

In our study, a comparison of chia with *O. tenuiflorum*, *M. longifolia*, *S. indicum*, and *S. lycopersicum* and the outgroup *A. thaliana* indicated that they share a common core of ~9,000 gene families with ~75,000 gene members. Chia shares more syntenic orthologs and collinearity (~9% colinear genes) with tomato than its distant relative *A. thaliana* (**Figure 2**). Interestingly, synteny comparisons between three publicly available chia genomes suggested a discrepancy in the naming of chia chromosomes (see **Supplementary Figure 4**). We propose consistent naming of the chia chromosomes from Sh1-6 based on their descending size and a reference genome nomenclature based on chromosome numbers and locations of the genes in the pseudochromosomes in our reference genome. We understand chia chromosome and gene nomenclature need a dialogue and collaborative work with the wider chia research community. Harmonizing genome and gene nomenclature is of high priority. It is required to develop a consistent reference framework for analyzing chia genomic and transcriptomic data and supporting functional genomics studies. We call upon the chia and Salvia research community to work together in proposing a consistent genome and gene nomenclature. It will help align the chia genomic data with the standards recommended by the AgBioData consortium (Harper et al., 2018), and adopt the Findable, Accessible, Interoperable, and Reusable (FAIR) data policy. In the near future, we would reach out to researchers to initiate this important work on chia genome nomenclature that will benefit the Salvia and chia genomics community, the plant genome databases, and INSDC data archive resources.

Finally, a careful comparison of all available chia genomes suggests that these sequenced genomes are from different individuals/accessions/lines. Thus, some differences in the genomic content and gene models between chia genomes are due to the cultivar differences. Combining all available chia genome sequences and mapping them against each other will not result in a single chia genome assembly of higher accuracy; it will generate a pan-genome assembly for different chia cultivars. Therefore, all individual chia genome and transcriptome sequences are highly relevant for future research and breeding of this understudied minor crop. Generating a comprehensive chia pangenome would require genome and transcriptome sequencing of additional chia cultivars, and these initial genome sequences will serve as references and anchors to capture the core and the accessory content of this species. The pangenome assembly of chia will be beneficial for understanding the genetic variation in this species and genus for improving chia yields and other agronomic and pharmacological important traits. This study also identified ~300,000 common SNP sites surveyed across the genus and ~15,000 SSR marker sites in the reference genome will help pursue genetic diversity studies and marker-assisted selection in chia breeding projects. The pyrimidine to pyrimidine (C↔T) SNP transitions is of particular interest because if the site is either in the transcribed coding or the regulatory region and is adjacent to a G, i.e., CpG site, it can be easily methylated and deaminated, which converts from T into a U, hence causing a G→A change that may lead to altered gene expression, function, or phenotype (Ashikawa, 2001).

In terms of economic and nutritional value, chia seed oil is an excellent plant-based source with one of the highest content of essential polyunsaturated fatty acids, including ~20% linoleic acid (LA), ~62% α-linolenic acid (ALA), and a minor fraction consisting of pentadecanoic acid, arachidonic acid (AA) and docosahexaenoic acid (DHA) (Segura-Campos et al., 2014). We identified (i) Δ12 and Δ15 desaturase genes involved in the biosynthesis of LA and ALA, respectively; (ii) three Δ6 desaturase genes responsible for the conversion of stearidonic acid (SDA- 18:4^Δ6,9,12,15^) from ALA and γ‐linolenic acid (GLA-18:3^Δ6,9,12^) from LA; and (iii) seven Δ6 elongase genes involved in elongation steps of SDA and GLA (**Figure 4A**). Previous reports from evening primrose, woodland anemone, black currant, and gibbous duckweed (Whitney et al., 2003; Song et al., 2010; Yan et al., 2013; Fu et al., 2017) showed the presence of Δ6 desaturase genes in plants. It is the first report on protein domain and homology-based identification of Δ6 desaturase and Δ6 elongase genes from chia. Here, we also report candidate genes involved in mucilage biosynthesis in seeds. The high amount of dietary fiber (30-40%) in chia seeds (Din et al., 2021) has increased its popularity in the medical field because it alleviates health disorders, such as type-2 diabetes and gastrointestinal (GI)‐tract‐related diseases (Khalid et al., 2023). A comprehensive analysis revealed that among the PUFA biosynthesis genes, exon skipping was the most prevalent splicing event, while in the seed mucilage biosynthesis pathway genes, intron retention emerged as the primary splicing event. Specifically, 17 PUFA genes and 36 seed mucilage biosynthesis pathway genes show splicing.

We expect that identifying candidate genes involved in PUFA, mucilage biosynthesis, and those coding for oleosin, caleosin, and steroleosin will aid in their functional characterization and potential application in improving chia seed size and nutritional quality. Previously, overexpression of *Brassica napus* oleosin genes in Arabidopsis seeds increased the seed size, weight, oleic acid, and linoleic acid content (Chen et al., 2019).

Besides the health benefit of PUFA and dietary fibers, plant proteins, including chia proteins, are known sources of bioactive peptides with potential anticancer, antioxidant, anti-inflammatory, antimicrobial, and antihypertensive properties (San Pablo-Osorio et al., 2019; Aguilar-Toalá and Liceaga, 2020; Ying et al., 2021; Aguilar-Toalá et al., 2022). Typically, proteins of plant origin have their intrinsic function in the plant body, but when consumed by other organisms, they could serve as a source of energy, signaling molecule, or bioactive peptides with potential health benefits. Previously chia seeds have been associated with ACE-inhibitory, antioxidant, antimicrobial, and hypocholesterolemic activities (Segura-Campos et al., 2013; Coelho et al., 2018). Thus, we performed an in-silico analysis of the predicted chia proteome for its potential to generate bioactive peptides relevant to human health. Our study suggested that 2707 genes that are highly expressed in the seed are likely to generate biopeptides with 18 different activities beneficial to human health (**Supplementary File 1**). Almost all of the antiaging peptides previously reported from chia (San Pablo-Osorio et al., 2019; Aguilar-Toalá and Liceaga, 2020; Aguilar-Toalá et al., 2022) were present in the seed storage proteins. We identified a Nictaba-domain containing lectin protein containing a biopeptide with a potential role in stomach mucosal membrane activity. Lectins from several genera of Lamiaceae, including the *Salvia* genus, have arisen considerable interest because of their potential use in detecting tumor cells (FPSB_1(2)288-299o.pdf; Fernández-Alonso et al., 2009; Duarte and Pérez, 2013).

Lectins are associated with plant development, cell signaling, and stress response and can act as mitogenic agents, biomarkers, and cytotoxic and insecticide proteins (Reyes-Montaño et al., 2018; Naithani et al., 2021b). We identified 98 members of the lectin gene family with a larger representation in the SD-RLK and legume-lectin subfamilies (**Figure 4A**). SD-RLKs are involved in signaling and development, etc (Vining et al., 2015; Naithani et al., 2021a), and many legume-lectins are associated with disease resistance (Gouget et al., 2006; Bouwmeester et al., 2011; Huang et al., 2013; Wang et al., 2014).

In addition to seeds, the leaves of chia plants are rich in secondary metabolites. The essential oil extracted from the shoot part of the chia plant is constituted mainly of sesquiterpenes, with caryophyllenes as the main constituents (Elshafie et al., 2018). Several pharmacological activities attributed to β-caryophyllene include antibiotic, antioxidant, anticarcinogenic, and local anesthetic (Legault and Pichette, 2007). We investigated Terpene Synthase (TPS) gene family in chia and identified 90 TPS genes classified under different clades (**Figure 4B**). We observed the maximum number of genes classified in TPS-a clade (37) followed by TPS-g (17) and TPS-b clade (14). Sesquiterpene and diterpene synthases mainly belong to TPS-a clade, and TPS-b and TPS-g clades primarily include monoterpene synthases (Chen et al., 2011).

In conclusion, this report on *Salvia hispanica* (chia) genome assembly (version #V1) provides nearly complete coverage of the gene space and contributes to developing genomic data resources. It is necessary to explore genetic variations in this under-studied minor (orphan) crop to support functional genomics (Naithani et al., 2017a; Naithani et al., 2021a), metabolic modeling (Dharmawardhana et al., 2013; Monaco et al., 2013; Naithani et al., 2014; Naithani et al., 2016), and its genetic improvement and breeding. This chia genome sequence, gene annotations, and transcriptome sequences could be a valuable resource to generate a future pan-genome of chia and support association studied on important crop traits (i.e., stress resilience, flowering time, nutritional quality, and yield) (Naithani et al., 2017b). While most plant genome reports are on inherent genome structure and gene function, this is the first report of a plant genome annotated for identifying biopeptides associated with improving human health. We expect that this study encourages other plant genomic scientists to consider linking and exploring the translational applications of plant omics studies to human health and nutrition.

## 4 Materials and methods

### 4.1 Plant material

The chia (*S. hispanica* L.) seeds from the second-generation inbred lines reported earlier were sown in 8-inch wide pots containing autoclaved soils and watered thoroughly under controlled greenhouse conditions. We harvested young leaf samples from 2-week-old seedlings pretreated in the dark for two days. Samples were immediately frozen in liquid nitrogen and stored at −80°C. Frozen tissue samples were shipped to Dovetail Genomics for genomic DNA extraction, sequencing, and assembly.

### 4.2 DNA extraction and genome sequencing

#### 4.2.1 Chicago library preparation and sequencing

A Chicago HighRise DNA sequencing library for genome scaffolding was prepared as described previously (Putnam et al., 2016). About 500 ng of high molecular weight gDNA with a mean fragment length = 75 was reconstituted into chromatin in *vitro* and fixed with formaldehyde. After digestion with DpnII, the 5' overhangs were filled with biotinylated nucleotides, and the free blunt ends were ligated. After ligation, crosslinks were reversed, and any proteins and unligated biotin were removed from the DNA fraction. The DNA was sheared to ~350 bp mean fragment size, and sequencing libraries were generated using Illumina-compatible adapters and NEBNext Ultra enzymes. Before PCR enrichment of each library, Biotin-containing fragments were isolated using streptavidin beads. The libraries were sequenced on an Illumina HiSeq X to produce 268 million 2x150 bp paired-end reads, which provided 97.85 x physical coverage of the genome (1-100 kb pairs).

#### 4.2.2 Dovetail HiC library preparation and sequencing

Two Dovetail HiC libraries were prepared as described previously (Lieberman-Aiden et al., 2009). Chromatin was fixed with formaldehyde in the nucleus for each library and then extracted. The rest of the steps were the same as described above for the Chicago library and sequencing. Sequencing produced 112 million, 2x150 bp for library 1 and 107 million, 2x150 bp for library 2; Together, the Dovetail HiC library reads provided 2,692.29 x physical coverage of the genome (10-10,000 kb pairs).

### 4.3 Genome assembly

A combination of 2x150bp paired-end (mean insert sizes ~363 bp and ~495 bp) reads generated from shotgun sequencing were used for constructing the *de novo* assembly. We used Meraculous 2.2.4 (diploid mode 1) (Chapman et al., 2011) with a kmer size 109. The input data consisted of 956 million read pairs sequenced from paired-end libraries (totaling 278.20 Gb). Reads were trimmed for quality, sequencing adapters, and mate-pair adapters using Trimmomatic (Bolger et al., 2014).

### 4.4 Scaffolding the assembly with HighRise

The input chia *de novo* assembly, shotgun reads, Chicago library reads, and Dovetail HiC library reads were used as input data for HiRise, a software pipeline designed specifically for using proximity ligation data to scaffold genome assemblies (Putnam et al., 2016). An iterative analysis was conducted. First, Shotgun and Chicago library sequences were aligned to the draft input assembly using a modified SNAP read mapper (SNAP | Essentials of Next Generation Sequencing). The separations of Chicago read pairs mapped within draft scaffolds were analyzed by HighRise to produce a likelihood model for the genomic distance between read pairs, and the model was used to identify and break putative misjoins, to score prospective joins, and make joins above a threshold. After aligning and scaffolding Chicago HighRise data, Dovetail HiC library sequences were aligned and scaffolded following the same method. After scaffolding, shotgun sequences were used to close gaps between contigs.

### 4.5 Organelle genome assemblies and annotation

The filtered and quality-assessed paired-end genome sequencing reads were used as inputs. The chloroplast and the mitochondrial genome were assembled using a combination of methods. First, the *de-novo* assemblies were carried out using the Novoplasty v2.7.2 (Dierckxsens et al., 2017) followed by reference-guided assemblies using the Geneious 11.1.5 (https://www.geneious.com/). Both *de-novo* and Geneious assemblies were again fed to Geneious to create a consensus assembly of the organelle genomes. The annotations were lifted from the publicly available chloroplast and mitochondrial genome assemblies available from the closest species of the Salvia genus and the *Arabidopsis thaliana*.

### 4.6 Genome assembly evaluation

We used the Benchmarking Universal Single-Copy Orthologs (BUSCO v5.3.2) approach to evaluate the accuracy and completeness of the genome assembly (Simão et al., 2015). BUSCO provides quantitative measures for the assessment of genome assembly based on evolutionarily informed expectations of gene content from near-universal single-copy orthologs. We evaluated both the genome assembly and the gene annotations by performing the BUSCO analysis on the Galaxy portal (The Galaxy Community, 2022) by selecting the MetaEuk gene predictor and other default parameters. For genome assessment, we compared the six largest scaffolds with previously published *de novo* transcriptome assembly derived using RNA-seq datasets for 13 tissues (Gupta et al., 2021) using GMAP (version 2013-11-27) (Wu and Watanabe, 2005).

### 4.7 Repeat identification andgenome masking

For the prediction of *de novo* repeats, we used the *de novo* prediction method of RepeatModeler (v1.0.11) (https://www.repeatmasker.org/RepeatModeler/) with the default parameters. In addition, we used repeat libraries from the six plant species (*Arabidopsis thaliana, Glycine max, Medicago truncatula, Lilium henryi, Triticum monococcum, Oryza sativa*) and combined them all to the *de novo* repeats identified by RepeatModeler. This combined library was used to mask the genome using a standalone version of RepeatMasker (v0406-09/28/2015) (http://www.repeatmasker.org/) with default parameters.

### 4.8 Gene prediction and functional annotation

Gene model prediction was performed using AUGUSTUS (v3.3.2) (Stanke et al., 2006). Peptide data sets from the five species, i.e., the model plant *Arabidopsis thaliana*, and four from the Lamiaceae family, *Salvia splendens*, *Ocimum tenuiflorum*, *Sesamum indicum*, *Mentha longifolia*, were used for generating the training gene set using the GenomeThreader and AUGUSTUS training pipeline. A trained data set and external hints generated from the previously published RNA-seq data from 13 tissue samples (**Table 2**) were used for the gene model prediction (Gupta et al., 2021). The gFACs v1.1.2 software was used to filter and get the final non-redundant gene models set (Caballero and Wegrzyn, 2019). The predicted gene model sequences were used to perform InterProScan that included Gene Ontology annotations (Jones et al., 2014). TargetP 2.0 and TMHMM 2.0 were used to predict organelle location and the presence of transmembrane domains, respectively (Krogh et al., 2001; Emanuelsson et al., 2007). Gene homology searches were performed against the NCBI-Viridiplantae database using BLASTP with an e-value cutoff of 1e-6 (Camacho et al., 2009).

### 4.9 Non-coding genes

The tRNAscan (Wang et al., 2014) identified the tRNA genes and their secondary structure with default parameters. RNAmmer 1.2 (Lagesen et al., 2007) was used to predict the ribosomal RNAs (rRNAs).

### 4.10 Synteny

Synteny comparison of the chia genome with the genomes of model plant *A. thaliana* (TAIR 10.26/CoGE ID 25869), and tomato (SL v3.0 with ITAG 3.2 gene modes/SGN v3/CoGE ID 13306) was performed using the SynMap from CoGE (Lyons and Freeling, 2008). The parameters used were Match score of 50, Match size of 5, Gap penalty of -1, Overlap window of 5, e-value 1e-05, and Max gap 25. SynMap with default parameters was also used to cross-check syntenic chromosomal mappings between ours and three previously published chia assemblies (NCBI; Wang et al., 2022; Alejo-Jacuinde et al., 2023; Li et al., 2023). The *Salvia* genome database section provides more details on the three genomes.

### 4.11 Transcriptome analyses

RNA-seq data from previously published (Gupta et al., 2021) 13 tissue types (**Table 2** and **Supplementary Table 8**) were used for differential gene expression analysis. RNA-seq reads were mapped to the chia reference genome using STAR (v2.7.2a), and gene counts were summarized by featureCounts (v2.0.0) (Dobin et al., 2013; Liao et al., 2014). Differential gene expression analysis was carried out using DESeq2 1.38.3 on NetworkAnalyst (v3.0) platform using one tissue versus all tissues strategy (Zhou et al., 2019).

### 4.12 Alternative splicing

Sequence reads from each RNA-Seq sample and replicates were aligned to the six largest scaffolds using the STAR aligner (Dobin et al., 2013). Alternative splicing events of chia genes were predicted across the genome using SpliceGrapher v0.2.5 pipeline (Rogers et al., 2012). Splice site-specific classifiers were built using the build classifiers.py script of SpliceGrapher using canonical (GT) and noncanonical (GC) donor sites and acceptor site (AG). Read alignment in SAM format was used as input for SpliceGrapher's sam_filter.py script to filter out false-positive sites. SpliceGrapher's Python scripts were used for the generation of depth files (sam_to_depths.py), splice graphs prediction (predict_graphs.py), generating statistics (splicegraph_statistics.py) from a set of splice graphs, gene-by-gene summary (genewise_statistics.py) of splicing events and splice graphs visualization (plotter.py).

### 4.13 Gene family analysis

Gene families were analyzed using the InParanoid software package (Östlund et al., 2010). InParanoid uses pair-wise reciprocal Blast analysis to look for sequence similarity and group similar genes in a cluster or family. Six species (*S. hispanica* (chia), *Mentha longifolia* (mentha/mint), *Ocimum sanctum* (basil), *Sesamum indicum* (Sesame), *Solanum lycopersicum* (tomato), and the model plant *Arabidopsis thaliana*) were compared. Since InParanoid only compares one species pair at a time, an algorithm to generate "super-clusters" or superfamilies, as described in Shulaev et al. (2011) was utilized. Gene families shared between the six species were sorted, and a gene member count-based matrix was created to analyze the gene gain and loss across these species using CAFÉ 2.0 (De Bie et al., 2006). A Newick taxonomy tree was retrieved from the National Center for Biotechnology Information (NCBI) taxonomy database for analysis. The most significant gene families were selected based on the p-value cutoff 0.01. The phylogenetic trees and the expression data was uploaded to the iTOL (Letunic and Bork, 2021) portal to generate figures.

### 4.14 Pathway analysis

The cDNAs and peptide sequences were analyzed against the KEGG (https://www.genome.jp/kegg) and Plant Reactome (https://plantreactome.gramene.org) to identify the pathway annotations for chia genes/proteins and map them to reactions and gene products (Kanehisa et al., 2016; Naithani et al., 2020). Mappings were appended with InterProScan and GO annotations to improve functional annotations. Subsequently, the inferred pathway annotations and the transcriptome data were used to analyze pathways of interest like polyunsaturated fatty acid biosynthesis, oil bodies, and biosynthesis of various dietary fiber constituents found in chia seeds.

### 4.15 Biopeptide discovery

We in-silico analyzed the presence of biopeptide signatures in the chia proteome that have potential to positively impact human health. This study used a library of curated biopeptides that are known to have a positive impact on human health. We used these bioactive peptides as a probe to identify similar sequence signature in chia proteins (predicted based on sequences). This was followed by querying the expression data of genes carrying such biopeptides. Thus, we predict that during gastrointestinal digestion, chia seed proteins (which contain sequence signatures of bioactive peptides) could potentially be fragmented into bioactive peptides by the actions of proteolytic enzymes (i.e., trypsin, elastase, different peptidases).

A set of 4,321 bioactive peptide sequences was downloaded from the BIOPEP-UWM database (Minkiewicz et al., 2019). Additional 20 known chia bioactive peptides (San Pablo-Osorio et al., 2019; Aguilar-Toalá and Liceaga, 2020; Aguilar-Toalá et al., 2022) were added to the reference library. This reference bioactive peptide library does not contain an exhaustive list, and a lot needs to be discovered via experiments and literature mining. A python script was written to search the chia amino acid sequences for times the sequences of each bioactive peptide were found. The script is available at https://github.com/Planteome/biopeptide_location (Elser and Jaiswal, 2023). This script takes two input files. (1) The curated biopeptide library, and (2) the fasta format protein sequences from the proteome. The curated library has information on the biopeptide_ID, biopeptid_name (biological role), peptide sequence, chemical mass, biological activity category (e.g. antibacterial, ACE inhibitor, anticancer, stimulating, binding, inhibitor, etc.). This step is followed by searching the curated biopeptide sequences for exact matches in the protein sequences listed in the proteome fasta file. Once the match is found, the best match results are provided in the output file that lists the id of the protein sequence in which the match was found, ID of the matched biopeptide, matched biopeptide_name, number of hits, biopeptide_sequence and location in the protein. At this time, the script does not look for the nearest protease digestion site to provide information on the putative digested peptide size.

### 4.16 Genetic variation

The whole genome shotgun paired-end sequence reads from *S. miltiorrhiza* (SRA: SRR2072001), *S. officinalis* (SRA: SRR6940041), *S. rosmarinus* (SRA: SRR6940042), *S. splendens* (SRA: SRR6382552) and *S. yangyii* (SRA: SRR6940082), and RNAseq reads from *S. divinorum* (SRA: SRR3716680), *S. pomifera* (SRNA: SRR2136651) and salba black and white from this project were used to call SNPs. The sequences from the SRA archive were pulled remotely and aligned with the salba white and black to the chia reference genome using the STAR v2.4.1d aligner (Dobin et al., 2013). The alignments were fed to the VarScan v2.3.9 for identifying the SNPs, including the indels (Koboldt et al., 2012). The identified variant SNP sites from each sample were further filtered for those with a minimum of 20 aligned read evidence. The simple sequence repeats (SSR), and sites were analyzed using the SSRIT tool (Temnykh et al., 2001) (https://archive.gramene.org/db/markers/ssrtool).

### 4.17 Salvia genome database

The JBrowse v1.0 (Buels et al., 2016) is used to serve the *Salvia* genome browser hosted on the *Salvia* genome database (SalviaGDB). Currently, the database carries genomes of *S. hispanica* from four sources, i.e. this report, Wang et al. (2022) accessed from the China National GeneBank DataBase under Bioproject no. CNP0002868 (Assembly ID: CNA0047366), Li et al. (2023) accessed from GenBank accession# PRJNA864090, and the NCBI RefSeq genome accession# PRJNA830713 published by the University of Melbourne, Australia. The polyploid genomes of *S. splendens* is from NCBI (genome assembly GCA_004379255.2), and *S. miltiorrhiza* is from the ENA accession# PRJNA287594) (Zhang et al., 2015; Dong et al., 2018). *S. bowleyana* and *S. rosmarinus* genomes were accessed from the Genome Warehouse of the National Genomics Data Center under accession number GWHASIU00000000 (https://bigd.big.ac.cn/gwh), and Figshare (https://doi.org/10.6084/m9.figshare.21443223.v1), respectively (Zheng et al., 2021; Han et al., 2023).

### 4.18 Data access

The sequence data described in this paper is available in the European Nucleotide Archive (ENA) at EMBL-EBI under project accession# PRJEB58694. The transcriptome sequence data is accessible from the EMBL-EBI ArrayExpress with an accession #E-MTAB-5515. The genome sequence and annotations are also accessible from the *Salvia* genomics database (SalviaGDB; https://salviagdb.org/). All data are provided for public access under the 'FAIR guiding principles for scientific data management and stewardship.' All raw and analyzed data is licensed under Attribution-NonCommercial-NoDerivatives 4.0 International.

## Data availability statement

The datasets presented in this study can be found in online repositories. The names of the repository/repositories and accession number(s) can be found in the article/**Supplementary Material**.

## Author contributions

PG: Data curation, Formal analysis, Investigation, Methodology, Project administration, Software, Validation, Visualization, Writing – original draft, Writing – review & editing. MG: Conceptualization, Data curation, Formal analysis, Funding acquisition, Investigation, Methodology, Project administration, Resources, Software, Supervision, Writing – original draft, Writing – review & editing. JE: Data curation, Formal analysis, Investigation, Methodology, Resources, Software, Visualization, Writing – original draft, Writing – review & editing. NA-B: Data curation, Formal analysis, Investigation, Methodology, Software, Validation, Writing – review & editing. RB: Data curation, Formal analysis, Investigation, Writing – review & editing. JP: Writing – review & editing, Methodology. EH: Writing – review & editing, Methodology. JP: Data curation, Formal analysis, Investigation, Methodology, Software, Visualization, Writing – review & editing. SN: Data curation, Formal analysis, Investigation, Methodology, Writing – original draft, Writing – review & editing. PJ: Validation, Visualization, Writing – original draft, Writing – review & editing, Conceptualization, Data curation, Formal analysis, Funding acquisition, Investigation, Methodology, Project administration, Resources, Supervision.
