# Supplementary material for: Reference genome of the nutrition-rich orphan crop chia (Salvia hispanica) and its implications for future breeding
Source: Front Plant Sci. 2023 Dec 14;14:1272966. doi: 10.3389/fpls.2023.1272966 (PMC10757625; doi:10.3389/fpls.2023.1272966)
Supplement: Supplementary file 1 [file DataSheet_1.zip › Supplementary Figures_updated.pdf]

a

k-mer Size	Error k-mer %	Modeled Non Repeat k-mers		Homozygous Peak	Estimated Heterozygous SNP %	Repeat k-mer %	Estimated Genome Size (Gbp)
		Heterozygous %	Homozygous %				
25	6.11	0.78	99.22	604.0	0.03	35.52	0.36
55	10.86	1.92	98.08	433.0	0.04	22.63	0.36
85	14.37	2.88	97.12	284.0	0.03	17.47	0.358
**109**	**16.75**	**4.16**	**95.84**	**175.0**	**0.04**	**15.47**	**0.356**

b

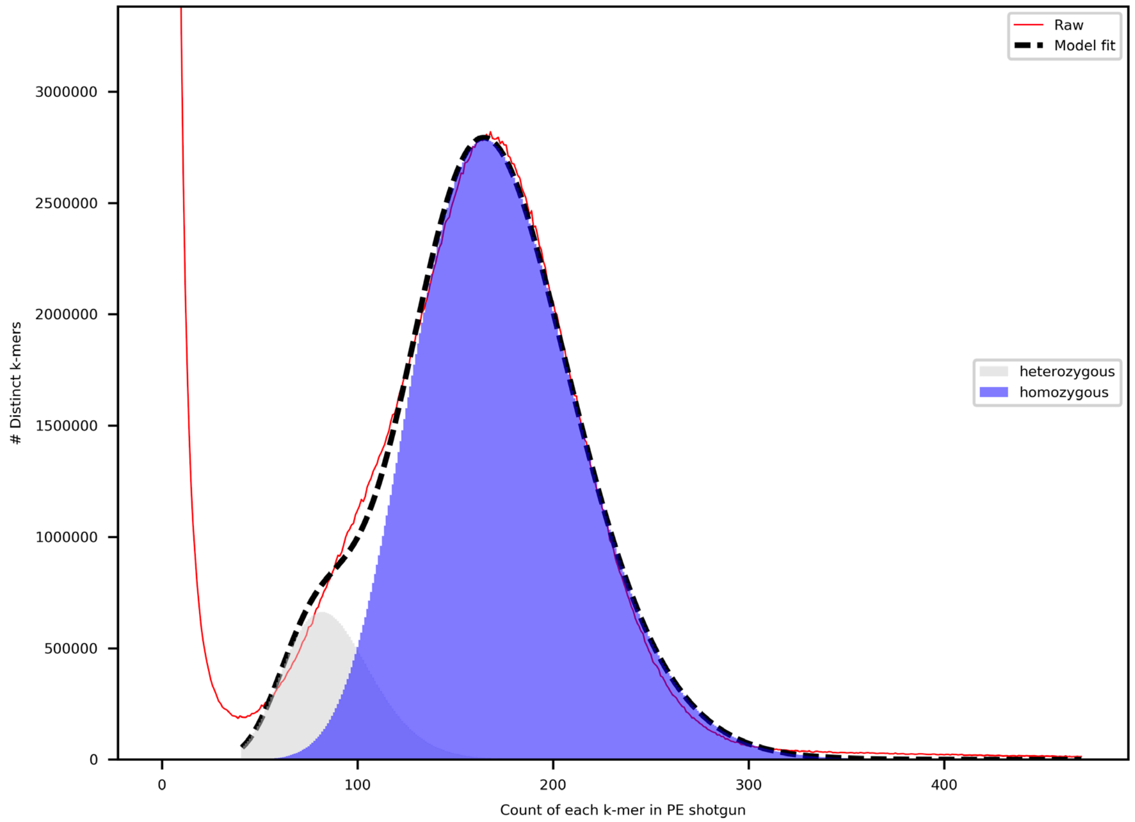

Supplementary Fig. 1: (a) k-mer analysis report; **(b)** Histogram of k-mer matrics. Red Line: Raw k-mer count; Dashed Black Line: Model fit over the raw k-mer count; Solid Blue: Homozygous negative binomial fit; Solid Grey: Heterozygous negative binomial fit.

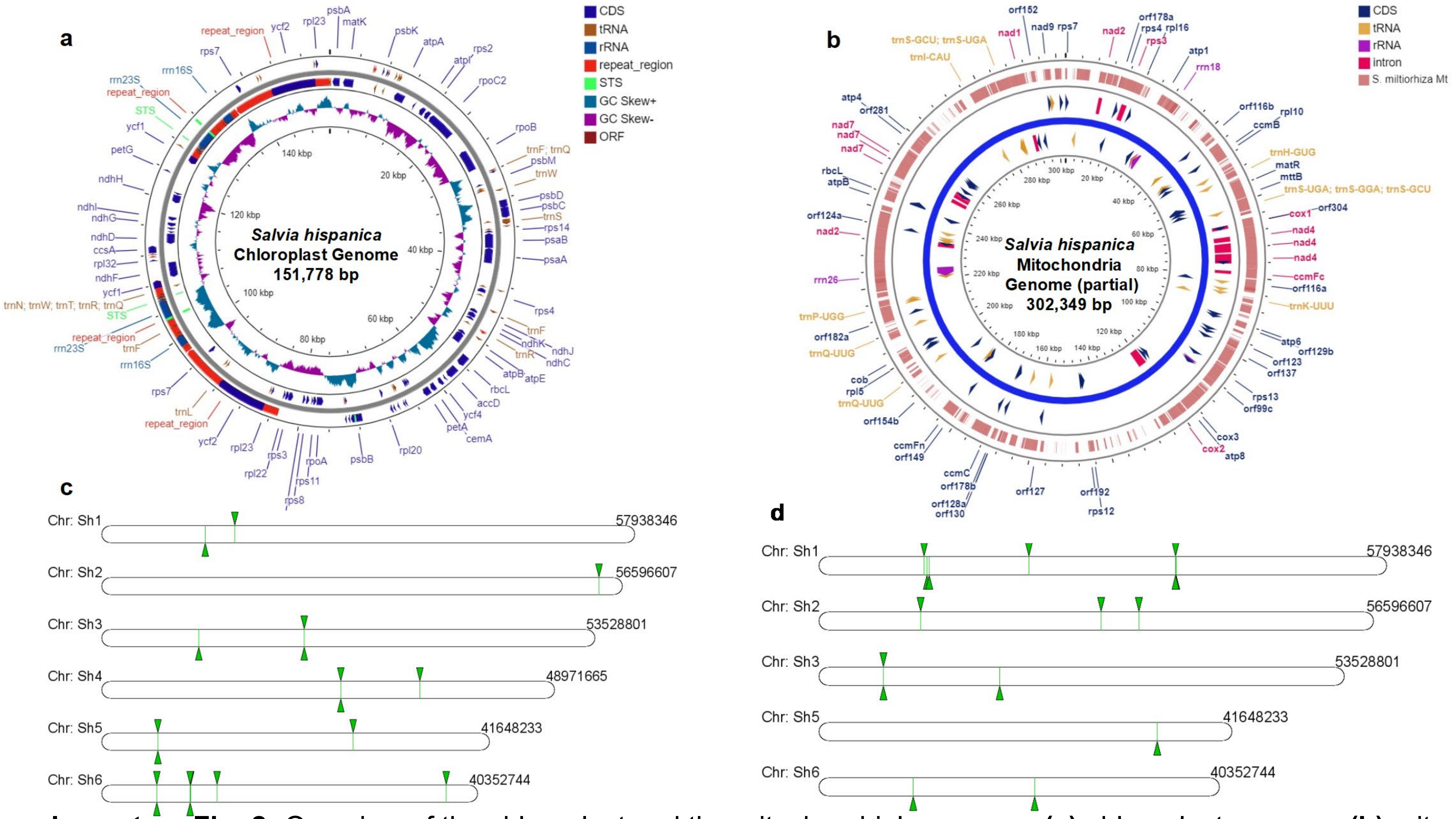

Supplementary Fig. 2: Overview of the chloroplast and the mitochondrial genomes. **(a)** chloroplast genome; **(b)** mitochondrial genome; **(c)** potential chloroplast genome insertion sites (green triangle) in the nuclear genome; **(d)** potential mitochondrial genome insertion sites (green triangle) in the nuclear genome

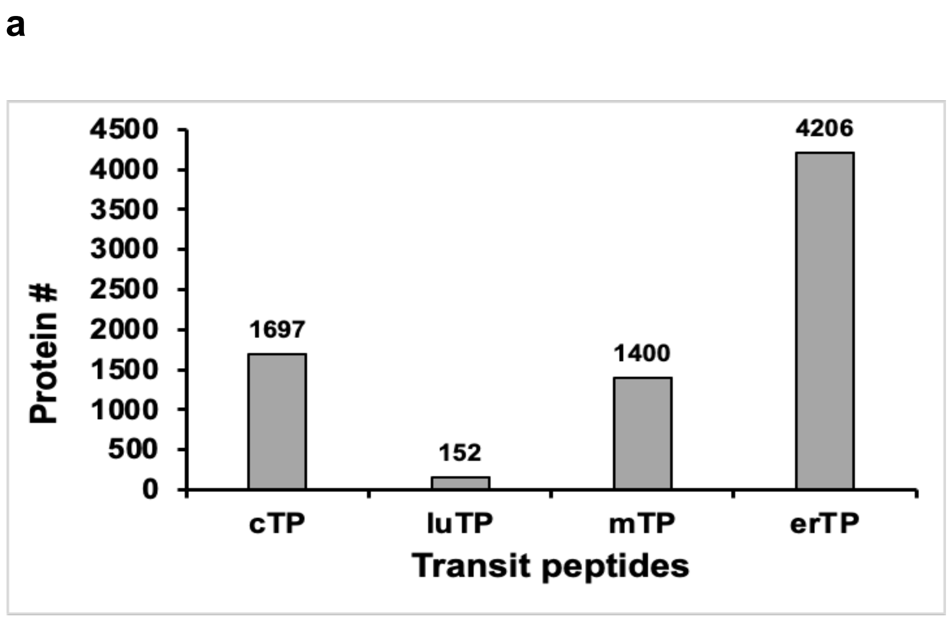

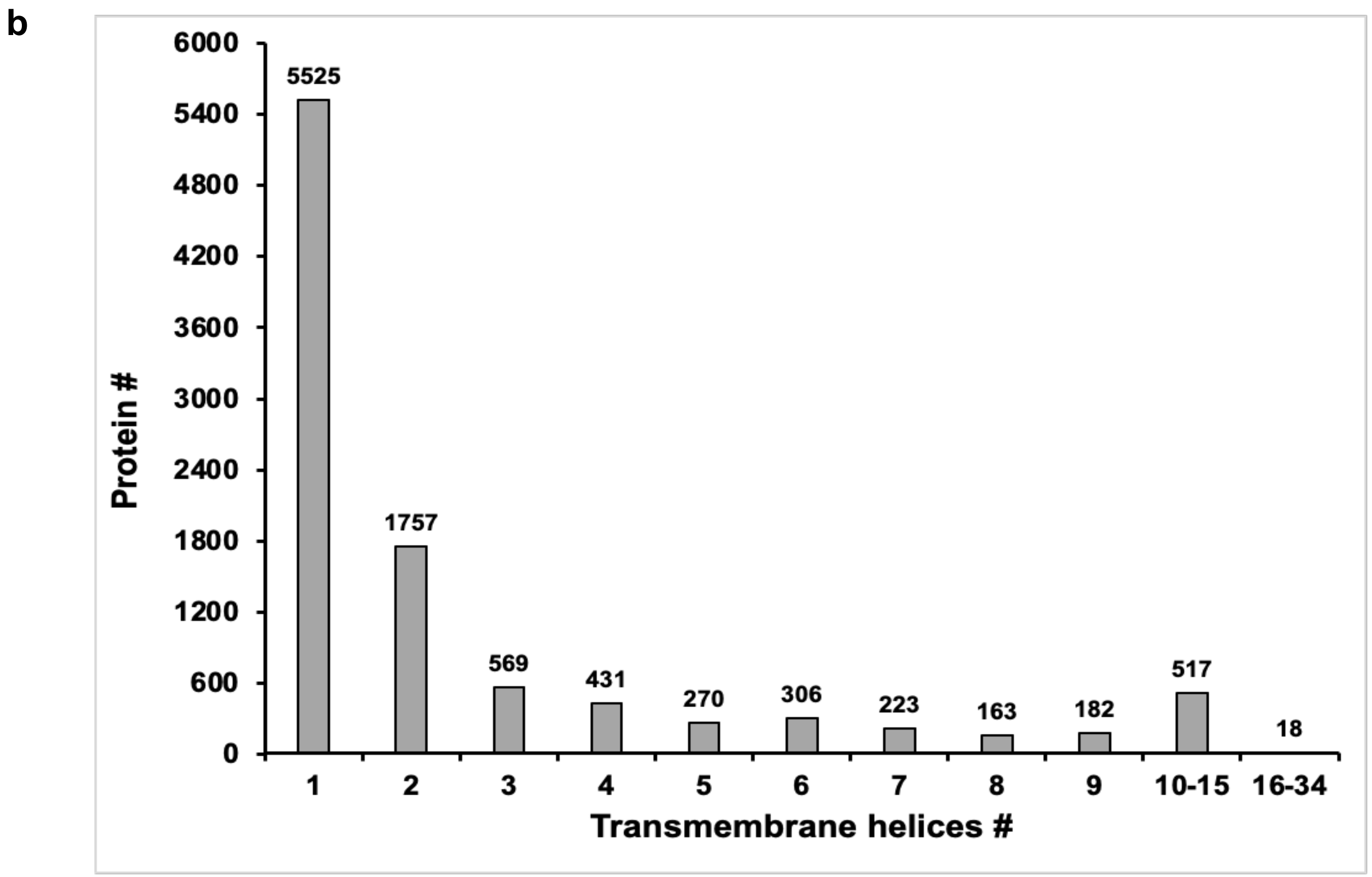

Supplementary Fig. 3: Functional annotation of Chia peptides. **(a)** Prediction of organelle localization signal sequences using TargetP; **(b)** Prediction of presence of transmembrane helices using TMHMM

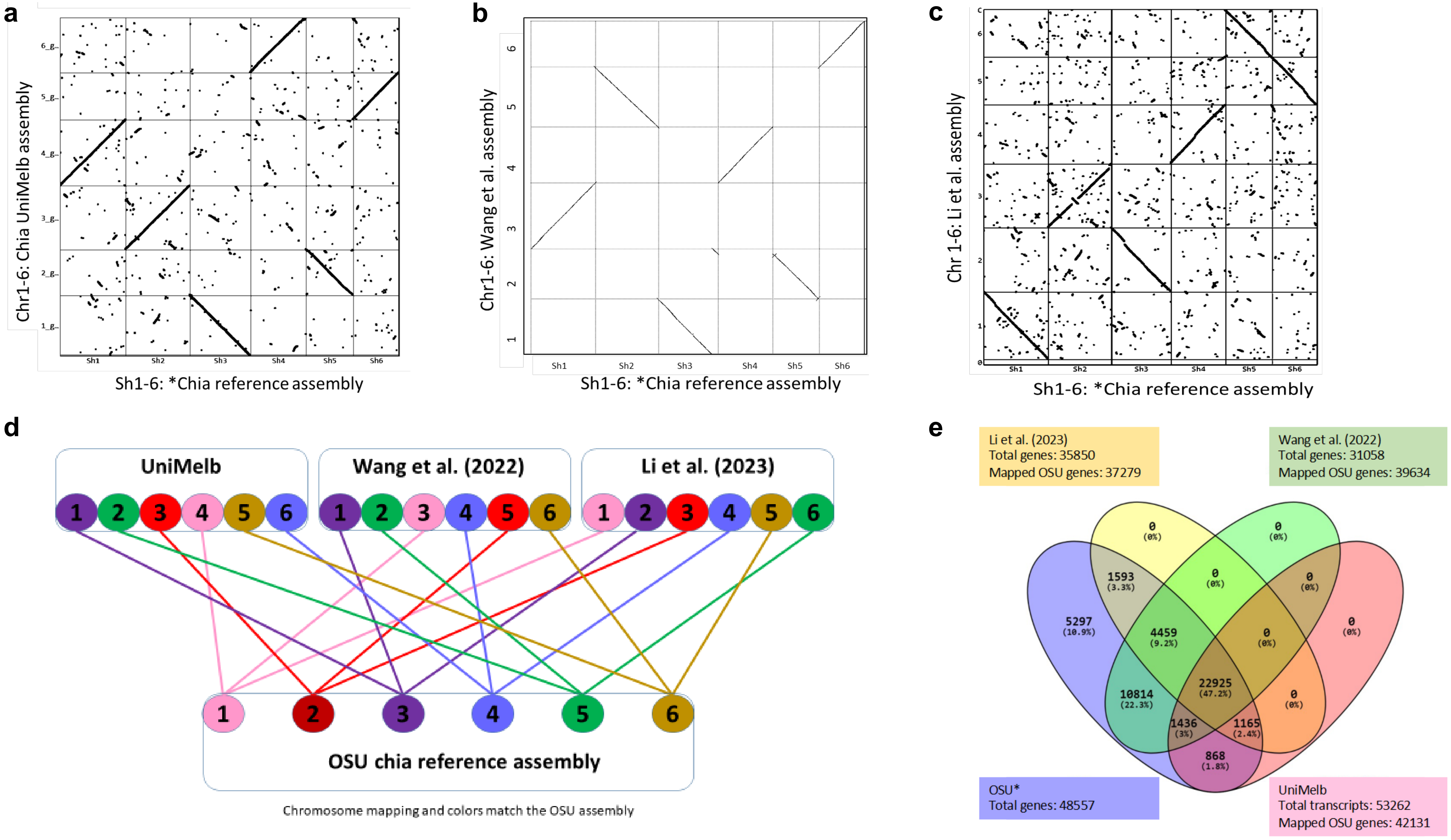

Supplementary Fig. 4 : Synteny and nomenclature comparison of chia genome assemblies. Chia reference assembly (*OSU) reported in this study, and **(a)** UniMelb assembly; **(b)** Wang et al. 2022; **(c)** Li et al. 2023; **(d)** chromosome nomenclature; **(e)** comparison of all OSU genes mapped to other chia genome annotations.

Genome Assembly	Assembled Genome Size	Assembled Genome in 6 chromosomes	Repeat content	Genes (protein coding)	Total transcripts	Mapped to								
						OSU*			Li et al. (2023)		Wang et al. (2022)		UniMelb	
						Genes	Genome	**de-novo transcripts	Genes	Genome	Genes	Genome	Transcripts	Genome
*OSU	303.6 MB	299.03 MB	42.09%	48090	54503			71417 (86.4%)	33619 (94%)	46843 (96%)	29129 (94%)	48474 (99.8%)	46628 (88%)	47272 (97.4%)
**OSU-denovo transcripts					82663	41912 (86.3%)	82145 (99.4%)		29280 (81.7%)	82461 (99.8%)	26862 (86.5%)	82396 (99.7%)	48914 (91.8%)	80865 (97.8%)
Li et al. (2023)	388 MB	361.7 MB	53.50%	35850	35850	37279 (77%)	34802 (97%)	67605 (82%)						
Wang et al. (2022)	347.6 MB	347.6 MB	45.70%	31058	31058	39634 (82%)	30298 (98%)	69246 (83.8%)						
UniMelb	321.31 MB	297.15 MB	NA	36995	53262	42131 (87%)	50836 (95%)	74730 (90.4)						

**OSU: genome annotation (this report); **OSU assembled transcripts reported in Gupta et al. (2021)*

Supplementary Fig. 5: Comparison of the chia genome assemblies and their annotated genes/transcripts, including the de-novo assembled transcriptome.

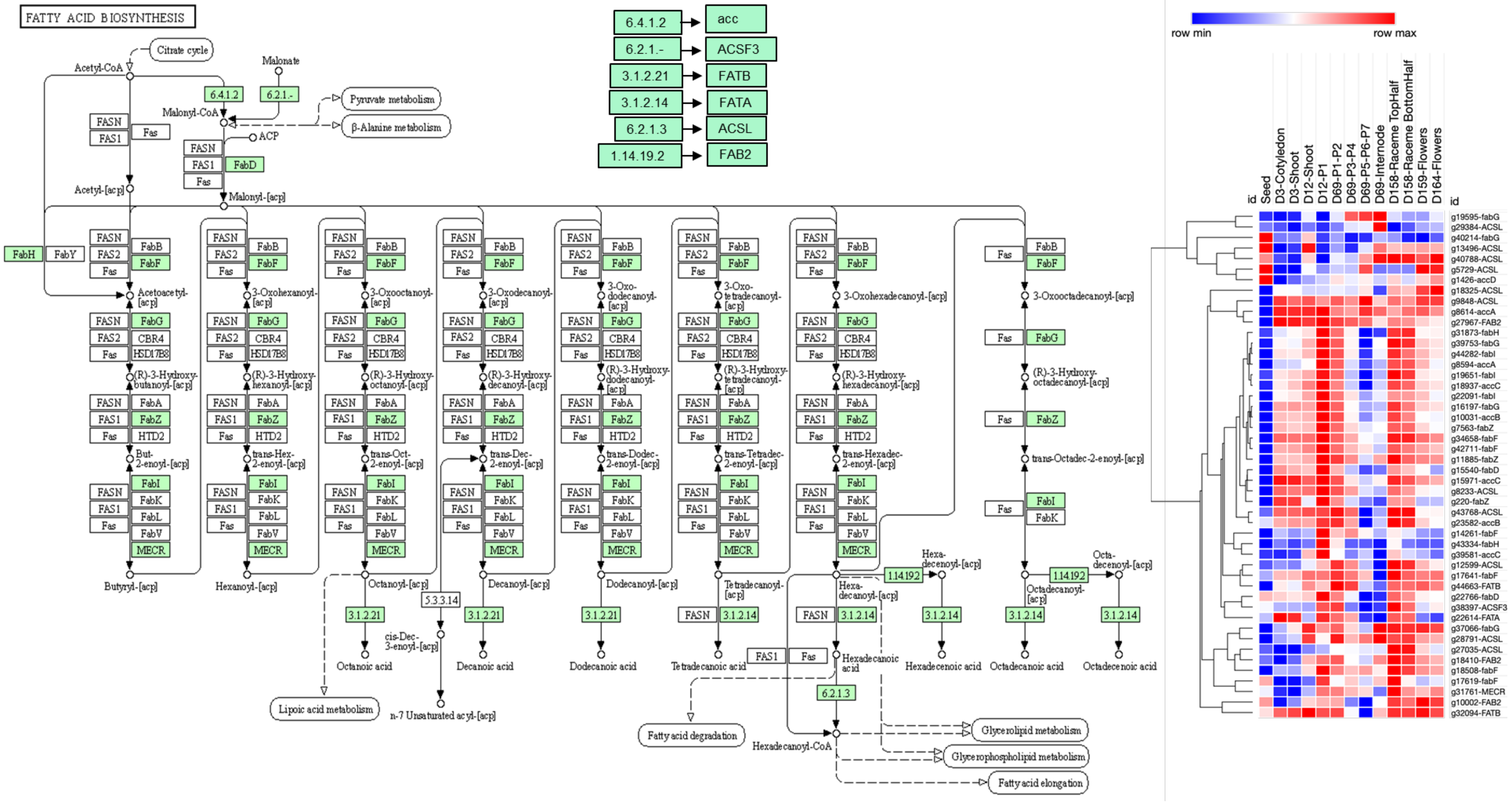

Supplementary Fig. 6: Pathway mapping and expression analysis of genes involved in fatty acid biosynthesis
