# Supplementary material for: Reference genome of the nutrition-rich orphan crop chia (Salvia hispanica) and its implications for future breeding
Source: Front Plant Sci. 2023 Dec 14;14:1272966. doi: 10.3389/fpls.2023.1272966 (PMC10757625; doi:10.3389/fpls.2023.1272966)
Supplement: Supplementary file 1 [file DataSheet_1.zip › Supplementary File 1.docx]

**Supplementary File 1:** Bioactive peptides with their potential roles. Type # include all the different type of peptides with similar roles. Instance # includes all the individual sites where the various all peptides with similar roles were discovered.

| **Peptide role** | **Chia peptides** | |
| --- | --- | --- |
|  | **Type #** | **Instance #** |
| ACE inhibitor | 708 | 5,939,132 |
| activating ubiquitin-mediated proteolysis | 3 | 152,461 |
| alpha-amylase inhibitor / Antidiabetic | 6 | 96 |
| alpha-glucosidase inhibitor / Antidiabetic | 27 | 300,413 |
| anorectic | 1 | 4 |
| anti-inflammatory | 22 | 51,174 |
| antiamnestic | 21 | 76,912 |
| antibacterial | 11 | 3,019 |
| anticancer | 7 | 6,803 |
| antidiabetic | 1 | 53 |
| antifungal | 3 | 7 |
| antioxidative | 471 | 977,148 |
| antithrombotic | 28 | 85,738 |
| antiviral | 7 | 363 |
| bacterial permease ligand | 3 | 64,231 |
| binding | 2 | 940 |
| CaMPDE inhibitor | 8 | 108,903 |
| celiac toxic | 5 | 400 |
| chemotactic | 6 | 1,961 |
| contracting | 5 | 332 |
| dipeptidyl peptidase III inhibitor / antihypertensive | 52 | 1,128,339 |
| dipeptidyl peptidase IV inhibitor / antidiabetic | 322 | 8,647,489 |
| embryotoxic | 3 | 2,269 |
| HMG-CoA reductase inhibitor | 5 | 8,807 |
| hypocholesterolemic | 1 | 19 |
| hypolipidemic | 2 | 33,134 |
| hypotensive / antihypertensive | 10 | 5,615 |
| immunomodulating | 38 | 38,770 |
| immunostimulating | 10 | 16,876 |
| inhibitor | 43 | 8,952 |
| neuropeptide | 12 | 95,325 |
| opioid | 34 | 6,471 |
| opioid agonist | 8 | 352 |
| opioid antagonist | 2 | 22 |
| pancreatic lipase inhibitor / anti-obesity | 2 | 12 |
| Protein Kinase C inhibitor / anticancer | 1 | 81 |
| regulating | 13 | 227,323 |
| renin inhibitor | 34 | 443,939 |
| stimulating | 25 | 717,985 |
| Elastase and hyaluronidase inhibitor | 7 | 11 |

List of biological roles that are known to be associated with the biopeptides identified in the proteins encoded by the upregulated genes expressed in the chia seed sample.

| **Biopeptide & Roles** |
| --- |
| ACE inhibitor |
| Antimicrobial |
| Beta-lactokinin |
| Coeliac toxic peptide |
| Dipeptidyl peptidase IV inhibitor (DPP IV inhibitor) |
| Dipeptidyl peptidase III inhibitor (DPP III inhibitor) |
| Elastase and hyaluronidase inhibitor (inhibit skin-aging) |
| Erythromycin inhibitor |
| Fragment of bovine beta-casein 191-193 |
| Immunostimulating peptide |
| Neuropeptide |
| Opioid peptide |
| PEP inhibitor |
| Peptide regulating ion flow |
| Peptide regulating phosphoinositol metabolism |
| peptide regulating the stomach mucosal membrane activity |
| Renin inhibitor |
| Tuftsin |

**Differentially expressed seed storage and lectin proteins with potential biopeptide activities**

**g21702, g21955, g21956, g31053, g32424, g32425**

Seed storage protein

Inhibit elastase and hyaluronidase (inhibit skin-aging)

Antimicrobial

**g38322**

SAWADEE HOMEODOMAIN HOMOLOGS 1 and 2 (SHH1 and SHH2) from plants

In Arabidopsis, SHH1 is a homeodomain protein required for DNA methylation.

ACE inhibitor

**g36678**

Napin/2s seed storage protein

ACE inhibitor

**g40103, g40104**

inhibit elastase and hyaluronidase (inhibit skin-aging)

Antimicrobial

Seed storage protein

Transmembrane (1x) longer C-terminus in cytoplasm

Nutrient reservoir activity (GO:0045735)

Mn binding sites

**g1424, g40776, g5726**

Nictaba lectin proteins

peptide regulating the stomach mucosal membrane activity

DPP-IV inhibitor

Antithrombotic peptide

ACE inhibitor

Ubiqitin-mediated proteolysis activating peptide

Antioxidative peptide

Renin inhibitor

CaMPDE inhibitor

Anxiolytic peptide

Glucose uptake stimulating peptide

Antioxidant peptide from marine bivalve (Mactra veneriformis)

Alpha-glucosidase inhibitor

DPP-III inhibitor

Anti-inflammatory peptide

Hypolipidemic peptide

Regulator of phosphoglycerate kinase activity

**g2634**

Legume-lectin protein

Peptide regulating phosphoinositol metabolism

Peptide regulating the stomach mucosal membrane activity

Renin inhibitor

Immunostimulating peptide

beta-lactokinin

Antithrombotic peptide

ACE inhibitor

Antioxidative peptide

DPP-IV inhibitor

DPP-III inhibitor

Kyotorphin

Hypolipidemic peptide

Alpha-glucosidase inhibitor
