# Supplementary material for: Reference genome of the nutrition-rich orphan crop chia (Salvia hispanica) and its implications for future breeding
Source: Front Plant Sci. 2023 Dec 14;14:1272966. doi: 10.3389/fpls.2023.1272966 (PMC10757625; doi:10.3389/fpls.2023.1272966)
Supplement: Supplementary file 1 [file DataSheet_1.zip › Supplementary Table 1.docx]

**Supplementary Table 1:** Length of six pseudomolecules (largest scaffolds) or Chromosomes

| **Pseudomolecules/**  **Chromosomes** | **Size (bp)** |
| --- | --- |
| Sh1 | 57,938,346 |
| Sh2 | 56,596,607 |
| Sh3 | 53,528,801 |
| Sh4 | 48,971,665 |
| Sh5 | 41,648,233 |
| Sh6 | 40,352,744 |
