# Supplementary material for: Reference genome of the nutrition-rich orphan crop chia (Salvia hispanica) and its implications for future breeding
Source: Front Plant Sci. 2023 Dec 14;14:1272966. doi: 10.3389/fpls.2023.1272966 (PMC10757625; doi:10.3389/fpls.2023.1272966)
Supplement: Supplementary file 1 [file DataSheet_1.zip › Supplementary Table 2.docx]

**Supplementary Table 2:** Summary of BUSCO evaluation

| **Database→** | **Viridiplantae db v10** | | **Eudicots db v10** | |
| --- | --- | --- | --- | --- |
| **Query→**  **Mapped BUSCO genes ↓** | **Genome** | **Genes** | **Genome** | **Genes** |
| Completeness | 97.6% | 94.4% | 95.6% | 90.6% |
| Complete | 415 | 401 | 2225 | 2108 |
| Complete and single copy | 381 | 373 | 1955 | 1865 |
| Complete and duplicated | 34 | 28 | 270 | 243 |
| Fragmented | 4 | 17 | 25 | 59 |
| Missing | 6 | 7 | 76 | 159 |
| Total BUSCO groups searched | 425 | | 2326 | |
