# Supplementary material for: Reference genome of the nutrition-rich orphan crop chia (Salvia hispanica) and its implications for future breeding
Source: Front Plant Sci. 2023 Dec 14;14:1272966. doi: 10.3389/fpls.2023.1272966 (PMC10757625; doi:10.3389/fpls.2023.1272966)
Supplement: Supplementary file 1 [file DataSheet_1.zip › Supplementary Table 3.docx]

**Supplementary Table 3:** Repetitive sequences found in the chia genome assembly

| **Repeat type** | **No. of elements** | **Percentage of the genome** |
| --- | --- | --- |
| LINEs | 1517 | 0.30 |
| LTR | 18567 | 6.01 |
| DNA elements | 12852 | 1.92 |
| Satellites | 3 | 0 |
| Simple repeats | 65446 | 0.93 |
| Low complexity | 13606 | 0.22 |
| Unclassified | 376159 | 32.82 |
