# Supplementary material for: Reference genome of the nutrition-rich orphan crop chia (Salvia hispanica) and its implications for future breeding
Source: Front Plant Sci. 2023 Dec 14;14:1272966. doi: 10.3389/fpls.2023.1272966 (PMC10757625; doi:10.3389/fpls.2023.1272966)
Supplement: Supplementary file 1 [file DataSheet_1.zip › Supplementary Table 4.docx]

**Supplementary Table 4:** Functional annotation of protein coding genes using InterProScan

| **Annotations** | **Number of protein coding genes** |
| --- | --- |
| InterPro | 33,710 |
| Gene Ontology | 18,620 |
| CDD | 10,517 |
| Gene3D | 9,425 |
| MobiDBLite | 7,402 |
| Pfam | 3,125 |
| Coils | 2,874 |
| SUPERFAMILY | 179 |
| ProSite Profiles | 103 |
| Hamap | 51 |
| ProSite Patterns | 11 |
| TIGRFAM | 9 |
| SMART | 6 |
| PRINTS | 4 |
| PIRSF | 3 |
| SFLD | 1 |
