# Supplementary material for: Reference genome of the nutrition-rich orphan crop chia (Salvia hispanica) and its implications for future breeding
Source: Front Plant Sci. 2023 Dec 14;14:1272966. doi: 10.3389/fpls.2023.1272966 (PMC10757625; doi:10.3389/fpls.2023.1272966)
Supplement: Supplementary file 1 [file DataSheet_1.zip › Supplementary Table 9.docx]

**Supplementary Table 10:** Genome-wide summary counts of spliced genes and splicing events. IR: Intron Retention; ES: Exon Skipping; Alt5’: Alternative 5’ splicing; Alt3’: Alternative 3’ splicing.

| **Chromosome** | **Total genes** | **Spliced genes** | **IR events** | **ES events** | **Alt5’ events** | **Alt3’ events** |
| --- | --- | --- | --- | --- | --- | --- |
| Sh1 | 9,688 | 4,329 | 3,719 | 2,887 | 971 | 1,321 |
| Sh2 | 10,723 | 4,850 | 4,210 | 2,935 | 1,028 | 1,334 |
| Sh3 | 7,715 | 3,093 | 2,747 | 2,057 | 714 | 977 |
| Sh4 | 7,150 | 3,093 | 2,734 | 1,680 | 641 | 807 |
| Sh5 | 6,811 | 3,078 | 2,595 | 2,043 | 664 | 891 |
| Sh6 | 6,656 | 2,848 | 2,455 | 1,615 | 669 | 816 |
